# Impact of Intrinsic Defects and Tungsten Doping on the Catalytic Properties of Two-Dimensional Cu_2_S

**DOI:** 10.1021/acsomega.6c03211

**Published:** 2026-05-26

**Authors:** Tarik Ouahrani, David Dell’Angelo, Mohammed Benaissa, Yasemin Oztekin Ciftci, Ángel Morales-García, Michael Badawi, Daniel Errandonea

**Affiliations:** † École Supérieure en Sciences Appliquées, ESSA-Tlemcen, BB 165 RP Bel Horizon, Tlemcen 13000, Algeria; ‡ Laboratoire de Physique Théorique, Université de Tlemcen, Tlemcen 13000, Algeria; § UCCS, CNRS, Université d’Artois, Faculté des Sciences Jean Perrin, Lens, 62307 Hauts-de-France, France; ∥ Laboratory of Materials Discovery, Unit of Research Materials and Renewable Energies, LEPM-URMER, Université de Tlemcen, Tlemcen 13000, Algeria; ⊥ 27079 Université de Rennes, CNRS, IPR (Institut de Physique de Rennes) - UMR 6251, F-35000 Rennes, France; # Department of Physics, Faculty of Science, 37511 Gazi University, 06500 Ankara, Turkey; ∇ Departament de Ciéncia de Materials i Química Física & Institut de Química Teórica i Computacional (IQTCUB), 16724 Universitat de Barcelona, c/Martí i Franquès 1-11, 08028 Barcelona, Spain; ° 137665 Université de Lorraine, CNRS, L2CM, F-54000 Nancy, France; ◆ Departamento de Física Aplicada - Instituto de Ciencia de Materiales, Matter at High Pressure (MALTA) Consolider Team, Universidad de Valencia, Edificio de Investigación, C/Dr. Moliner 50, Burjassot, 46100 Valencia, Spain

## Abstract

Understanding how intrinsic defects govern dopant activity is central to the rational design of catalytic two-dimensional materials. Here, combining first-principles defect thermodynamics with hydrogen adsorption descriptors, we investigate the defect landscape and catalytic behavior of a Cu_2_S monolayer. Our calculations show that sulfur-on-copper antisites (S_Cu_) are the most thermodynamically favorable intrinsic defects, with defect formation energies ranging from approximately −0.12 to 0.35 eV depending on the chemical potential environment. In contrast, copper vacancies (V_Cu_) exhibit higher defect formation energies in the range of 0.45 to 0.85 eV, but become increasingly stabilized under Cu-poor conditions. Importantly, we demonstrate that thermodynamic stability does not directly correlate with catalytic activity. Although S_Cu_ is energetically favored, it shows weak hydrogen binding with a hydrogen adsorption free energy of Δ*G*_H*_ = −0.66 eV, whereas V_Cu_-based configurations significantly improve hydrogen adsorption toward more optimal values. Single tungsten substitution at Cu sites is found to preferentially stabilize a V_Cu_+W defect complex with a formation energy of approximately 0.28 eV, which modifies the electronic structure by introducing acceptor-like states near the valence band edge while avoiding strongly localized defect levels that may hinder charge transport. This defect-mediated electronic restructuring leads to near-thermoneutral hydrogen adsorption, with Δ*G*_H*_ improving from −0.66 eV for pristine Cu_2_S to −0.08 eV for the V_Cu_+W complex. Overall, these results highlight how the interplay between intrinsic defects and extrinsic dopants can be exploited to tune the electronic structure and catalytic behavior of Cu_2_S, providing general design principles for defect-engineered electrocatalysts for the hydrogen evolution reaction.

## Graphical abstract

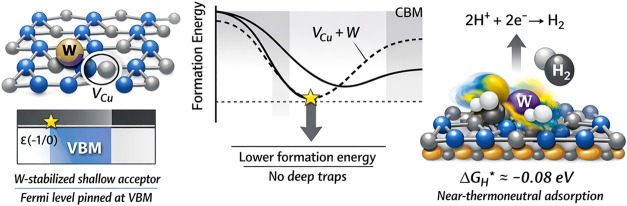

## Introduction

The urgent need to shift from fossil fuel-based energy systems to sustainable alternatives has driven significant research into hydrogen evolution reaction (HER) electrocatalysts, as hydrogen is regarded as a clean, high-energy-density energy carrier.^1^ Two-dimensional (2D) materials have emerged as promising candidates for HER platforms due to their exposed active sites, tunable electronic structures, and large surface areas.^2^ Various strategies have been proposed to enhance or activate catalytic sites in 2D materials,^3–6^ such as elemental doping, single-atom anchoring, and strain engineering. However, doping remains a challenging approach due to its dependence on factors like site selection, dopant stability, and defect formation energies, despite its potential to significantly improve catalytic activity.^2^ A successful doping strategy requires careful selection of dopant elements that introduce the desired electronic states without compromising structural integrity, as well as a comprehensive point-defect analysis to identify electronically active and energetically favorable sites. To develop durable and efficient HER catalysts, these rational design principles are essential. In summary, effective doping strategies must balance the choice of dopants with an in-depth understanding of defect chemistry, ensuring both enhanced catalytic performance and structural stability.^7^

Transition-metal chalcogenides, particularly copper sulfide (Cu_2_S), exhibit significant potential for nanoelectronic and optoelectronic applications due to their tunable band structures.^8^ Although bulk Cu_2_S is not a naturally layered van der Waals material, its low-dimensional forms have been widely reported in both experimental and theoretical studies. In particular, Cu_2_S has been successfully synthesized as thin films, nanorods, nanosheets, and quantum-dot-based heterostructures, demonstrating its strong morphological versatility.^9–11^ Recent experimental works have further shown that defect engineering and morphology control in copper sulfides can significantly enhance their photocatalytic and electrocatalytic performance.^12–14^ Cu_2_S monolayers are especially promising for **p**-type semiconducting applications and energy-related devices because they are made from low-cost elements, offer high carrier mobility, and have a moderate band gap.^15^ However, like many 2D materials, the electronic properties of Cu_2_S are strongly influenced by intrinsic defects, which can alter its optical and transport characteristics.^16–19^ Defects such as vacancies and antisites are common in 2D Cu_2_S and play a crucial role in determining carrier type and mobility during synthesis. Specifically, sulfur vacancies (V_S_), copper vacancies (V_Cu_), and copper interstitials (Cu_i_) create deep acceptor-like states near the conduction band, trapping electrons and reducing electron conduction.^20^

Cu_2_S is particularly sensitive to intrinsic defects due to its buckled structure, where Cu atoms occupy a central plane, and S atoms are displaced above and below it, creating nonequivalent atomic sites. This geometry enhances the localization of defect-induced electronic states, amplifying their impact on the electronic properties of the material. Consequently, the intrinsic defect landscape often impedes the achievement of stable **p**-type conduction, limiting the practical implementation of Cu_2_S in electronic devices. Developing effective strategies to suppress or passivate these defects is therefore essential, as it could substantially broaden the applicability of Cu_2_S monolayers in FinFET-based 2D transistors and photovoltaic devices.

Substitutional doping offers a viable strategy to overcome the limitations imposed by unfavorable deep trap levels in semiconducting materials.^21,22^ In strongly oxidized systems such as the structure considered here, replacing Cu atoms with metal dopants—rather than substituting S atoms—can generate acceptor states near the valence band maximum (VBM). These states can effectively compensate deep acceptor-like intrinsic defects and thereby improve electronic performance.^23,24^

Although elemental doping is widely used to enhance the catalytic activity of two-dimensional materials, its success is often limited by issues such as dopant stability, site selectivity, and complex interactions with intrinsic defects. Consequently, achieving improved catalytic behavior demands a comprehensive understanding of defect physics, particularly carrier compensation mechanisms and defect-induced electronic states. In this work, we tackle this challenge by integrating first-principles defect thermodynamics with hydrogen adsorption energetics to clarify the influence of intrinsic defects and tungsten substitution on the properties of Cu_2_S monolayers.

This compensation mechanism promotes stable hole conduction while suppressing unintended deep acceptor–induced **p**-type conductivity. Consequently, substitutional doping at Cu sites represents an effective approach to enhance electrical performance and achieve controlled **p**-type behavior.^23^ Substituting Cu with suitable dopants provides a rational pathway to tailor both the electronic structure and catalytic activity of Cu_2_S.^25^

Transition-metal doping is a well-established strategy to tune the electronic and catalytic properties of two-dimensional chalcogenides. In particular, WS_2_ has been widely studied due to its chemical stability and enhanced hydrogen evolution activity upon transition-metal substitution. Both experimental and theoretical works have shown that W-based systems can effectively modulate the electronic structure and optimize hydrogen adsorption energetics.^26^ Motivated by these results, tungsten is used here as a representative and experimentally relevant transition-metal dopant to study defect–dopant interactions in Cu_2_S. Under appropriate growth conditions, substitutional W (W_Cu_) can be thermodynamically stable in Cu_2_S. The interaction between W_Cu_ and copper vacancies (V_Cu_) leads to the formation of a defect complex that modifies the electronic structure by introducing acceptor-like states near the valence band maximum (VBM), while avoiding the formation of deep trap states within the band gap. This defect–dopant interaction can effectively compensate intrinsic defect levels and improve carrier redistribution, thereby promoting more stable hole transport in Cu_2_S.

Moreover, strong hybridization between W 5*d* orbitals and S 3*p* states induces significant charge redistribution at the surface,^27,28^ which modulates the hydrogen adsorption free energy toward near-thermoneutral values. This electronic restructuring improves the interaction between the active sites and hydrogen intermediates, thereby favoring the hydrogen evolution reaction (HER). The W_Cu_ defect thus provides a coherent coupling between defect-induced electronic states and surface reactivity, arising from the combined effects of vacancy formation and transition-metal substitution. This coupling enables a tunable adsorption landscape for hydrogen without destabilizing the host lattice. In this study, we focus on the electrocatalytic hydrogen evolution reaction (HER) under electrochemical conditions. The term “catalytic performance” refers specifically to the intrinsic activity toward HER, which is evaluated using the Gibbs free energy of hydrogen adsorption (Δ*G*_H*_), a widely accepted descriptor of electrocatalytic efficiency.

## Computational Methodology

The Vienna *Ab initio* Simulation Package (VASP)^29^ was employed for all density functional theory (DFT) calculations. The projector augmented-wave (PAW) method^30^ (version 5.4) was used to model the interaction between core and valence electrons, while the exchange-correlation effects were treated within the generalized gradient approximation (GGA) using the Perdew–Burke–Ernzerhof (PBE) functional.^31^ The GGA + *U* method^32^ was applied, where *U* represents the Hubbard correction in Dudarev’s formalism, with *U* values of 5 eV for the *d*-orbitals of the Cu atom (see Figure S1 in the Supporting Information). To achieve total-energy convergence within 1 meV/atom, a plane-wave energy cutoff of 500 eV was used.

All calculations were performed within a spin-polarized framework, allowing for a fully self-consistent treatment of spin degrees of freedom and accurately capturing the potential magnetic properties of the system originating from the Cu atom. The on-site Coulomb interaction was included using the GGA + *U* method within the Dudarev formalism,^33^ ensuring proper treatment of the localized Cu 3*d* orbital.^34^ This approach enabled the recovery of the experimental band gap of 1.2 eV within a small difference of 0.1 eV.^35,36^ To ensure numerical accuracy, systematic convergence tests were conducted before proceeding with the main calculations. To verify the consistency of our results, we additionally carried out calculations using the Heyd–Scuseria–Ernzerhof (HSE06) hybrid functional on the unit cell of the studied compound. The calculated band gap is 1 eV (see the Supporting Information), which agrees well with the GGA + *U* result. The discrepancy between the two methods is approximately 0.1 eV, confirming that the GGA + *U* approach provides a reliable estimate of the band gap. To model doping, a substitutional concentration of 0.66% was employed, corresponding to the replacement of one host atom by a dopant in a 150-atom 5 × 5 × 1 supercell (see convergence test in Figure S2 in the Supporting Information). This concentration represents a balance between computational feasibility and physical realism. By introducing a single dopant atom into a relatively large supercell, a sufficiently dilute regime is achieved, minimizing artificial dopant–dopant interactions arising from periodic boundary conditions and allowing the dopant to be treated as an isolated defect. At the same time, this supercell size remains computationally manageable for hybrid-functional or DFT+*U* calculations, ensuring a realistic description of dilute doping without excessive computational cost. Electronic self-consistency was achieved with a strict convergence criterion of 10^–7^ eV for the total energy between successive self-consistent field iterations. To avoid spurious interactions between vertical periodic images, a vacuum region of at least 20 Å was included along the out-of-plane direction to model the Cu_2_S monolayer. Defect calculations were performed using the PBE + *U* functional on the 150-atom 5 × 5 × 1 supercell. A **k**-point convergence test was performed for the defect supercell, showing that a Γ-4 × 4 × 1 grid ensures total energy convergence within 1 meV^37^ (see convergence test in Figure S3 in the Supporting Information). van der Waals interactions were considered using the DFT-D3 method,^38,39^ and the lattice dynamics was calculated with the phonopy package.^40,41^

The hydrogen evolution reaction (HER) in acidic media occurs through proton–electron transfer steps at the catalyst surface. The primary descriptor influencing HER activity is the Gibbs free energy of hydrogen adsorption, Δ*G*_H*_, which measures the thermodynamic favorability of hydrogen binding to the active sites.^42^

The hydrogen adsorption energy, Δ*E*_H*_, was calculated as^42^

1 $$\Delta E_{H*}=E_{\mathrm{slab}+H}-E_{\mathrm{slab}}-\frac{1}{2}E_{H_{2}}$$

where *E*_slab+H_ and *E*_slab_ denote the total energies of the surface with and without an adsorbed hydrogen atom, respectively, and *E*_H_2__ is the total energy of an isolated hydrogen molecule in the gas phase.

To obtain the Gibbs free energy of adsorption, zero-point energy (ZPE) and entropy corrections were included

2 $$\Delta G_{H*}=\Delta E_{H*}+\Delta E_{\mathrm{ZPE}}-T\Delta S$$

where $\Delta E_{\mathrm{ZPE}}=E_{\mathrm{ZPE}}\left(H^{*}\right)-\frac{1}{2}E_{\mathrm{ZPE}}\left(H_{2}\right)$ , and $\Delta S=S\left(H^{*}\right)-\frac{1}{2}S\left(H_{2}\right)$ . The entropy of adsorbed hydrogen was assumed to be negligible compared to that of gas-phase hydrogen.

Within the computational hydrogen electrode (CHE) model, the combined proton–electron chemical potential in acidic conditions is referenced to half of the hydrogen molecule

3 $$\mu \left(H^{+}+e^{-}\right)=\frac{1}{2}E_{H_{2}}$$

An ideal HER catalyst is characterized by Δ*G*_H*_ ≈ 0 eV, corresponding to a balanced hydrogen adsorption strength that facilitates both hydrogen adsorption and desorption.

## Results and Discussion

### Crystal Structures and Stability

The Cu_2_S monolayer under investigation can crystallize into two distinct phases: the orthorhombic described by space group *Pmma* and the tetragonal described by space group *P42*_1_2.^35,36^ It exhibits a highly anisotropic in-plane atomic arrangement. Our calculations indicate that the tetragonal structure is the more stable one, as shown in Figures S4 and S5 of the Supporting Information. This conclusion aligns with previous findings.^35,36^ In the tetragonal phase, the Cu–S network naturally arranges into two distinct crystallographic directions. Along the *a*-axis, the atoms adopt a zigzag-like edge configuration (see Figure 1). The presence of both zigzag and armchair motifs underscores the low-dimensional nature of the material, highlighting its intrinsic structural anisotropy. After full structural relaxation, the optimized lattice parameter for the Cu_2_S monolayer was determined to be *a* = 5.48 Å, which is in good agreement with previous theoretical studies,^36,43^ confirming the reliability of our computational approach.

**Figure 1:**
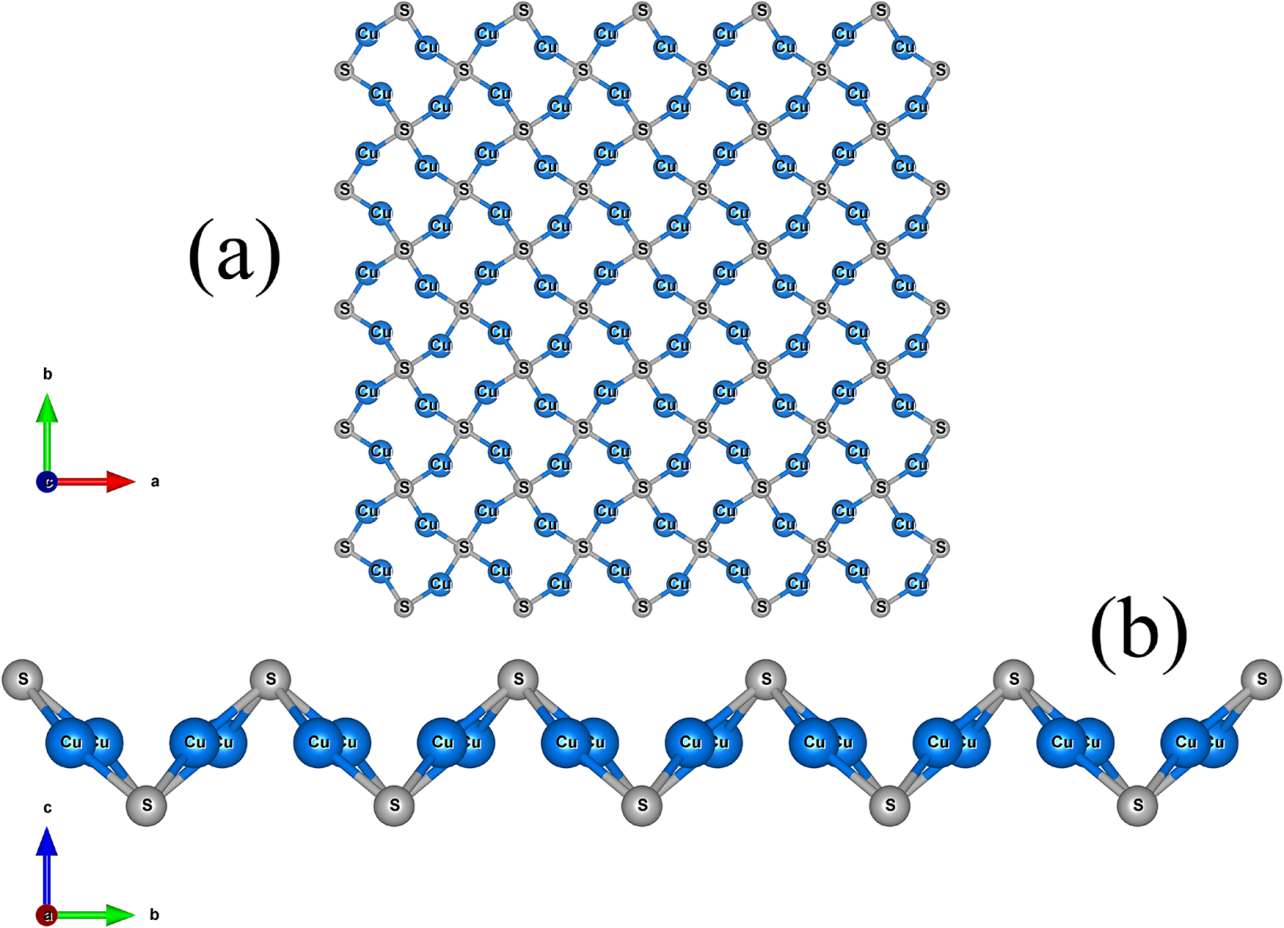
Top (a) and side (b) views of the optimized structure of the Cu_2_S monolayer shown as a 5 × 5 × 1 supercell. The top view reveals the anisotropic atomic arrangement associated with zigzag and armchair directions, while the side view confirms the planar geometry of the monolayer. Copper and sulfur atoms are depicted as blue and gray spheres, respectively.

The phonon dispersion spectrum of the tetragonal Cu_2_S monolayer, shown in Figure 2(a), provides important insights into its dynamical stability. The phonon branches are plotted along the high-symmetry directions of the Brillouin zone (X−Γ–M–Y). Notably, no imaginary phonon frequencies are observed across the entire wave-vector range, indicating that the optimized Cu_2_S monolayer is dynamically stable and represents a true minimum on the potential energy surface. The spectrum reveals that the low-frequency region is dominated by acoustic and low-energy optical modes, which are primarily associated with vibrations of Cu atoms. In contrast, sulfur vibrations, which involve bond-stretching motions within the Cu–S framework, give rise to the high-frequency optical modes. The partial phonon density of states shown in Figure 2(b) further supports this interpretation, with S atoms dominating in the high-frequency range and Cu contributions concentrated at lower frequencies.

**Figure 2:**
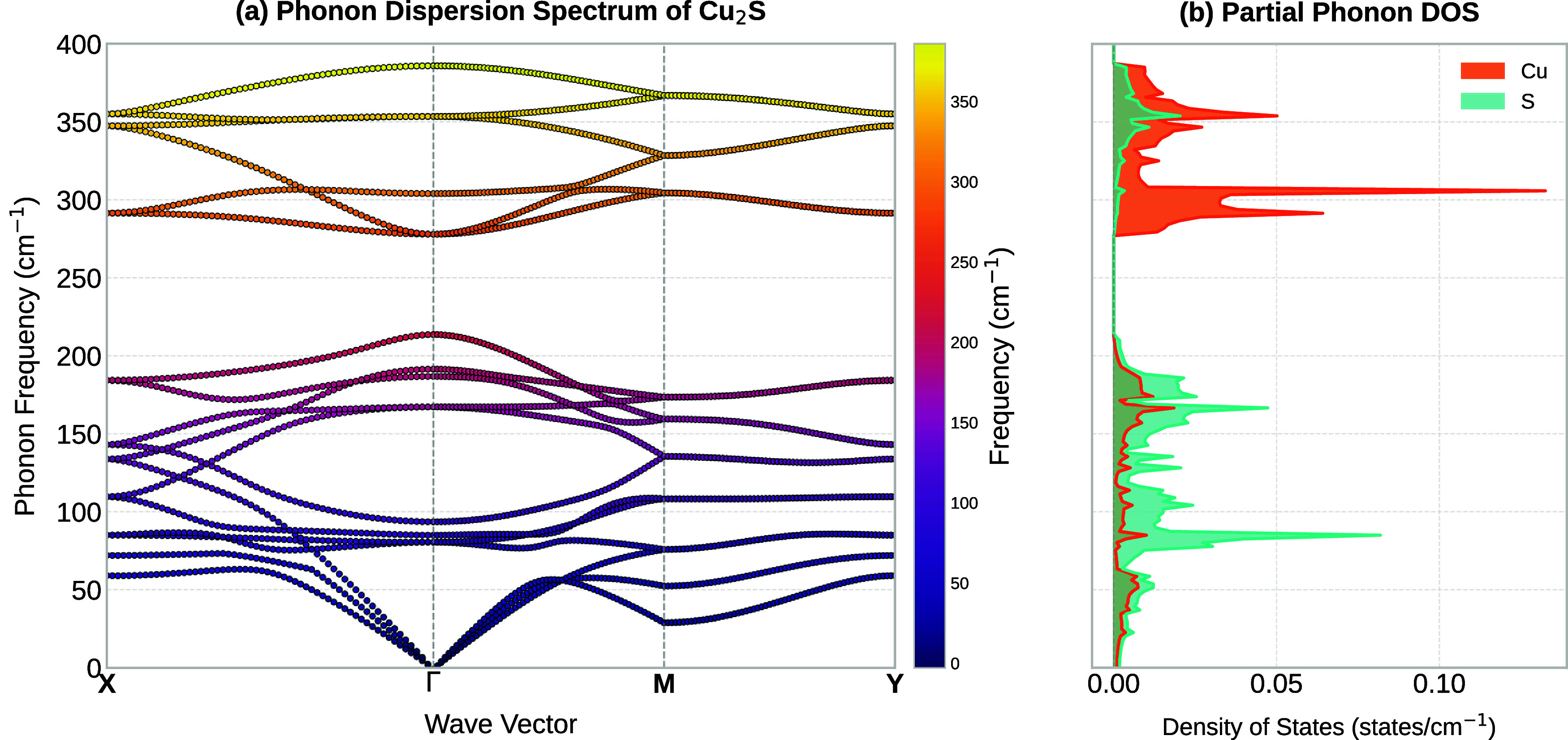
(a) Phonon dispersion and (b) phonon density of states of the tetragonal Cu_2_S single layer.

### Analysis of Defect Structures

The various defect configurations considered in this study are depicted in Figure 3. To obtain physically meaningful formation energies, the multistep process for calculating point-defect properties in two-dimensional materials requires a careful definition of chemical potentials, large supercell models, and accurate treatment of charged defects. The procedure starts with the following equation^44^

4 $$\Delta E_{f}\left(D^{q}\right)=E_{\mathrm{tot}}\left(D^{q}\right)-E_{\mathrm{tot}}\left(\mathrm{pristine}\right)-\sum_{i}n_{i}\mu_{i}+q\left(E_{F}+E_{\mathrm{VBM}}\right)+E_{\mathrm{corr}}$$

**Figure 3:**
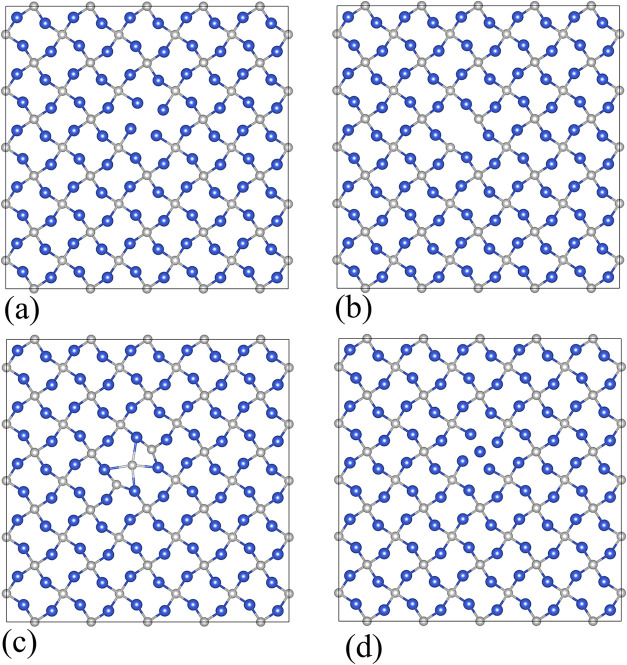
Schematic representation of the four different defect structures considered for the Cu_2_S monolayer, modeled using a 5 × 5 × 1 supercell: (a) sulfur vacancy (V_S_), (b) copper vacancy (V_Cu_), (c) antisite S_Cu_ (a sulfur atom occupying a copper site), and (d) antisite Cu_S_ (a copper atom occupying a sulfur site). Gray and blue spheres represent Cu and S atoms, respectively.

eq 4 represents the formation energy of a point defect *D* in charge state *q*, where *E*_tot_(*D* ^*q*^) and *E*_tot_(pristine) are the total energies of the defective and pristine supercells, respectively. The quantity *n*_*i*_ represents the number of atoms of species *i* added (*n*_*i*_ > 0) or removed (*n*_*i*_ < 0) from the supercell, with the corresponding atomic chemical potential denoted by μ_*i*_. The valence-band maximum *E*_VBM_ of the pristine system serves as the reference for the Fermi level *E*_F_.

Let us begin by determining the quantity (∑_*i*_ *n*_*i*_μ_*i*_). The chemical potentials of copper (μ_Cu_) and sulfur (μ_S_) are constrained by the formation energy of Cu_2_S

5 $$2\mu_{\mathrm{Cu}}+\mu_{S}=-0.825\mathrm{eV}$$

where Δ*H*_*f*_(Cu_2_S) = – 0.825 eV is the calculated formation enthalpy (GGA + *U*). Consequently, we found that to ensure thermodynamic stability against competing (Cu_6_S_4_, Cu_12_S_6_, Cu_2_S_2_, Cu and S) phases, the chemical potentials must satisfy the following conditions

6 $$\mu_{\mathrm{Cu}}<0,\mathrm{(prevention}\;\mathrm{of}\;\mathrm{Cu}\;\mathrm{precipitation)}$$

7 $$\mu_{S}<0,\mathrm{(prevention}\;\mathrm{of}\;S\;\mathrm{precipitation)}$$

8 $$\mu_{\mathrm{Cu}}>-0.325\mathrm{eV},\left(\mathrm{stability}\;\mathrm{against}\;{\mathrm{Cu}}_{2}S_{2}\;\mathrm{formation}\right)$$

9 $$6\mu_{\mathrm{Cu}}+4\mu_{S}<\Delta H_{f}\left({\mathrm{Cu}}_{6}S_{4}\right),({\mathrm{Cu}}_{6}S_{4}\;\mathrm{formation}\;\mathrm{limit)}$$

10 $$12\mu_{\mathrm{Cu}}+6\mu_{S}<\Delta H_{f}\left({\mathrm{Cu}}_{12}S_{6}\right),({\mathrm{Cu}}_{12}S_{6}\;\mathrm{formation}\;\mathrm{limit)}$$

11 $$2\mu_{\mathrm{Cu}}+2\mu_{S}<\Delta H_{f}\left({\mathrm{Cu}}_{2}S_{2}\right),({\mathrm{Cu}}_{2}S_{2}\;\mathrm{formation}\;\mathrm{limit)}$$

In particular, the Cu-poor limit is determined by the Cu_2_S_2_ stability condition

12 $$\mu_{\mathrm{Cu}}+\mu_{S}<-0.5$$

Using eq 5, μ_S_ = −0. 825 – 2 μ_Cu_, we obtain

13 $$\mu_{\mathrm{Cu}}+\left(-0.825-2\mu_{\mathrm{Cu}}\right)<-0.5$$

14 $$-\mu_{\mathrm{Cu}}-0.825<-0.5$$

15 $$\mu_{\mathrm{Cu}}>-0.325\;\mathrm{eV}$$

Solving these constraints yields the stable chemical potential range

16 $$-0.325\;\mathrm{eV}<\mu_{\mathrm{Cu}}<0\;\mathrm{eV}\;\mathrm{and}\;-0.825\;\mathrm{eV}<\mu_{S}<0\;\mathrm{eV}$$

The two extreme conditions considered in this study are (see Figure 4)­

- Cu-rich/S-poor: μ_Cu_ = 0 eV, μ_S_ = −0.825 eV
- Cu-poor/S-rich: μ_Cu_ ≈ −0.33 eV, μ_S_ ≈ −0.165 eV

**Figure 4:**
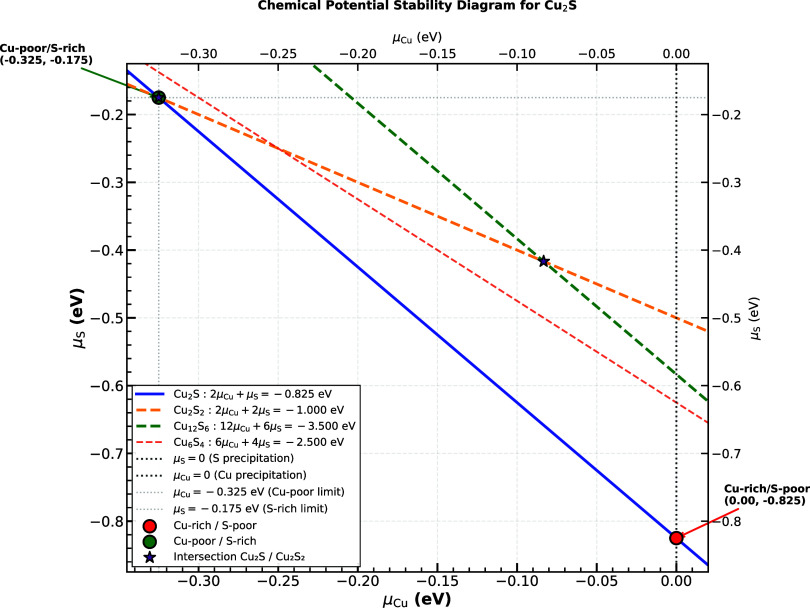
DFT-calculated chemical potential stability diagram of Cu_2_S (GGA+*U*). The shaded region shows the thermodynamic stability domain of Cu_2_S, bounded by the Cu_2_S formation line (blue solid), the Cu_2_S_2_ (orange dashed) and Cu_12_S_6_ (green dashed) competition limits, and the elemental stability boundaries (μ_Cu_ < 0, μ_S_ < 0). The extreme points correspond to Cu-rich/S-poor (μ_Cu_ = 0 eV, μ_S_ = −0.825 eV, red point) and Cu-poor/S-rich (μ_Cu_ ≈ −0.33 eV, μ_S_ ≈ −0.165 eV, green point) conditions. The Cu_6_S_4_ limit (red dashed) is shown for reference. The shaded region (or line segment) represents the thermodynamic stability domain of Cu_2_S along the equilibrium condition.

To evaluate the remaining quantities in eq 4 and analyze the intrinsic point defects in the Cu_2_S structure, we considered four defect configurations: the sulfur vacancy (V_S_), the copper vacancy (V_Cu_), the sulfur-on-copper antisite (S_Cu_), and the copper-on-sulfur antisite (Cu_S_).

Interstitial defect configurations were not considered in this study, as they typically exhibit high (positive) defect formation energies in two-dimensional chalcogenide systems and thus possess negligible thermodynamic stability.^45^ It is also important to emphasize that supercell-size convergence is essential for an accurate description of point defects. Insufficiently large supercells can introduce spurious interactions between periodically repeated defects, leading to artificial shifts in defect formation energies and charge-transition levels. Therefore, systematic supercell-size convergence tests were carried out prior to the main calculations, with the results presented in Figure S2 of the Supporting Information. Moreover, charged defects in periodic supercells can induce artificial shifts in the reference electrostatic potential and generate spurious electrostatic interactions between periodic images. To correct for these effects, the correction term *E*_corr_ was evaluated to account for the periodic interaction between charges in doped systems. A 2D-dimensional implementation of the Freysoldt–Neugebauer–Van de Walle (FNV) correction scheme,^46^ adapted for slab geometries, was employed to obtain reliable values of *E*_corr_.

To capture the possible ionization states of each defect under varying electronic conditions, the defect charge state *q* was examined over a broad range, namely *q* = −2, −1, 0, + 1, and +2.^2^ This approach enables a comprehensive evaluation of defect stability and charge-dependent behavior within the host material. Full methodological details and corresponding results are provided in the Supporting Information. Using this framework, the defect formation energy Δ*E*_*f*_(*D* ^*q*^) was calculated for each charge state. For clarity and to facilitate tracking of the computational procedure, all relevant quantities and results are summarized in Table S1 of the Supporting Information. In the main manuscript, we focus on the charge-neutral case (*q* = 0) for clarity, while results for all other charge states are presented in the Supporting Information (see Figure 5, Figure S6, and Tables S2 and S3).

**Figure 5:**
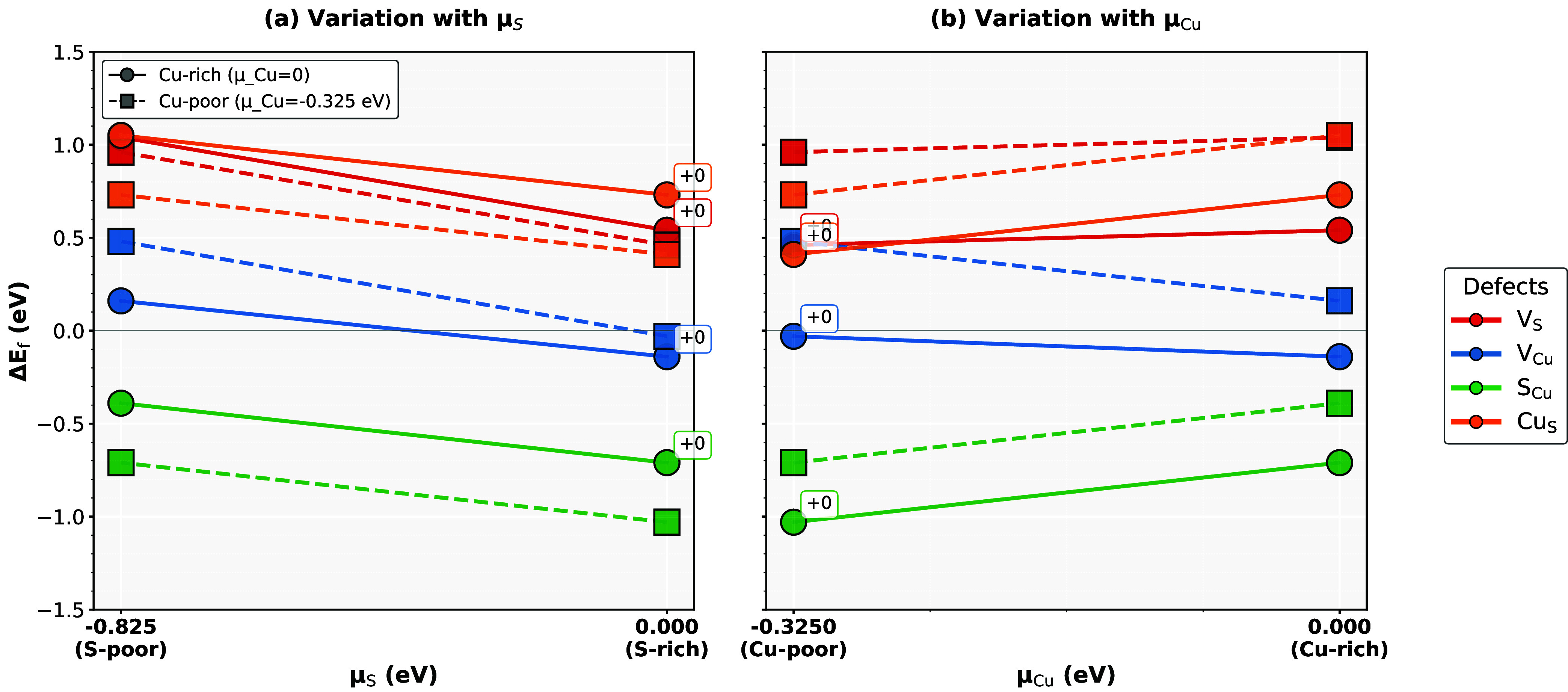
Point-defect formation energy diagrams showing the formation energies Δ*E*_*f*_ of intrinsic point defects in the Cu2S monolayer as a function of the Fermi level *E*_*F*_, referenced to the valence band maximum (VBM). Panel (a) presents the formation energies under Cu-rich conditions with Δ*μ*Cu = 0, while panel (b) corresponds to S-rich conditions with Δ*μ*_S_ = 0. Thicker solid lines highlight the neutral charge states (*q* = 0).

As shown in Figure 6, variations in the chemical potentials primarily affect the absolute values of defect formation energies, while leaving their slopes with respect to *E*_*F*_ unchanged. This illustrates that the intercept is governed by the growth environment, whereas the gradient is determined solely by the defect charge state (*q*). Consequently, the relative stability ranking of intrinsic defects remains largely consistent across different chemical potential conditions.

**Figure 6:**
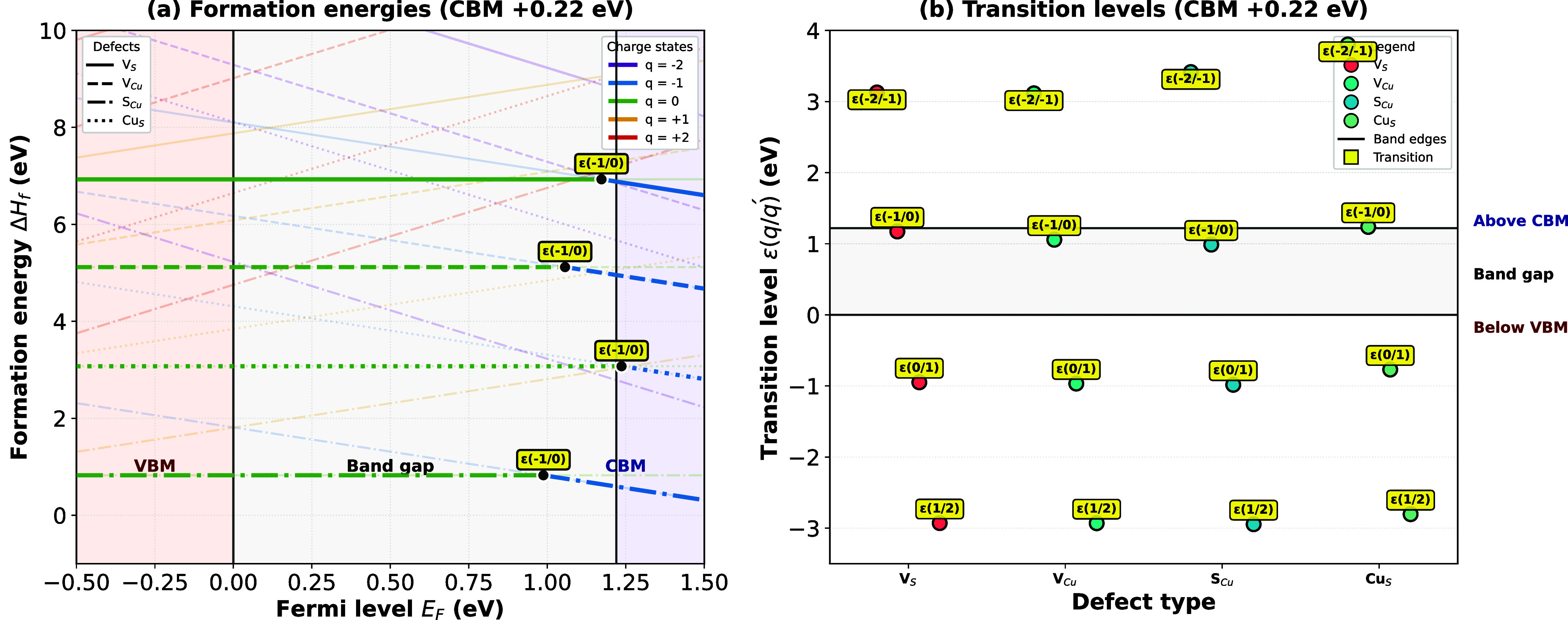
(a) Formation energies of intrinsic point defects in the Cu_2_S monolayer as a function of the Fermi level under stoichiometric conditions, highlighting the positions of thermodynamic charge transition levels. The slope of each line corresponds to the charge state of the defect, and the lowest-energy envelope determines the thermodynamically stable charge state at a given Fermi level. (b) The thermodynamic charge transition levels *ε*(*q*/*q*′) of intrinsic defects within the Cu_2_S monolayer are represented in relation to the valence band maximum (VBM at 0 eV). Transitions located within the shaded gray region correspond to deep defect states situated within the band gap.

Figure 6 shows that the sulfur antisite (S_Cu_, green line) consistently exhibits the lowest defect formation energy, remaining negative under both S-rich and Cu-rich environments. This indicates that S_Cu_ is energetically favorable and likely the dominant intrinsic defect during synthesis. In contrast, the sulfur vacancy (V_S_, red line) and copper antisite (Cu_S_, orange line) have significantly higher positive formation energies (above 0.4 eV), reflecting their relative instability and low likelihood of spontaneous formation. Notably, under Cu-rich conditions [panel (a) of Figure 6], the copper vacancy (V_Cu_, blue line) becomes increasingly stabilized as the environment shifts toward S-rich conditions, even reaching negative formation energies.

These findings highlight the critical influence of intrinsic defect chemistry on the electronic properties of the Cu_2_S monolayer, beyond the effects of extrinsic growth conditions. The prevalence of low-energy acceptor-type defects over a wide Fermi-level range underscores the material’s intrinsic tendency toward **p**-type behavior, consistent with experimental observations in Cu-based sulfide systems.

### Thermodynamic Charge Transition Levels

Thermodynamic charge transition levels serve as a key indicator for evaluating the effectiveness of metal doping. The dopability of a material is determined by the position of these levels relative to the band edges, which reveals whether a dopant can generate free carriers or will be compensated by intrinsic defects. The thermodynamic charge transition levels, *ε*(*q*/*q*′), are defined as the points where the formation-energy lines of different charge states intersect. The calculated results are shown in Figure 6.

17 $$\varepsilon \left(q/q^{\prime }\right)=\frac{\Delta E_{f}\left(D^{q},E_{F}=0\right)-\Delta E_{f}\left(D^{q\prime },E_{F}=0\right)}{q^{\prime }-q}$$

Figure 6 provides a comprehensive overview of the intrinsic defect behavior that governs the electronic properties of the Cu_2_S monolayer under stoichiometric conditions. In panel (a), the defect formation energies are plotted as a function of the Fermi level, enabling direct identification of the most stable charge state for each defect across the band gap. The slopes of the formation-energy lines correspond to the respective defect charge states, while the lower envelope indicates the thermodynamically preferred configuration at a given *E*_*F*_. All intrinsic defects considered exhibit similar behavior, introducing deep trap states associated with donor-like charge transitions located at or above the conduction band minimum (CBM). Notably, the charge transition level of the S_Cu_ defect lies at the edge of the CBM, whereas those of the other defects are positioned deeper within the conduction band, as illustrated in Figure 6(a).

Consequently, these defects act as dominant compensating centers that trap carriers and suppress electron transport in the system. Although they exhibit donor-like charge states, their thermodynamic charge-transition levels are near or above the conduction band minimum (CBM), rendering them electronically inactive for carrier generation under realistic conditions. As a result, electron occupation of these defect states does not lead to the formation of free charges; therefore, these defects contribute negligibly to **p**-type conductivity. Instead, they pin the Fermi level and compensate donor states, thereby limiting the effectiveness of **n**-type doping through a parasitic compensation mechanism.

### The Effect of W Doping on the Hydrogen Evolution Reaction Property

As discussed above, both pristine Cu_2_S and its defective structures introduce defect states within the band gap; however, these states are predominantly deep, acting as carrier traps and thus limiting efficient carrier generation and transport. To counteract this trend, we draw inspiration from WS_2_, where experimental studies have shown that Ir doping induces a robust **n**-to-**p**-type conversion.^47^ In particular, degenerate Ir doping in WS_2_ significantly enhances charge transport and electrical conductivity, resulting in a **p** ^+^-WS_2_ semiconductor with superior HER performance compared to pristine **n**-type WS_2_. For instance, the **p** ^+^-WS_2_-9.8 catalyst exhibited markedly reduced overpotentials and favorable Tafel slopes across a broad pH range, achieving 92 mV vs RHE and 33 mV dec^–1^.

Motivated by this strategy, we considered introducing several transition-metal dopants into the Cu_2_S monolayer, including Ag, Au, Zn, Cd, Hg, Co, and W, which possess electronic configurations that can, in principle, interact with Cu sites and modulate the electronic structure. In selecting a representative dopant for this study, we focused on general practical considerations relevant to catalytic materials design, such as chemical stability, environmental compatibility, and relevance to experimentally accessible systems. Within this context, tungsten (W) was chosen as a representative dopant due to its well-known chemical robustness and resistance to degradation under electrochemical conditions in both acidic and alkaline environments.^48^ Importantly, W is not assumed to be superior to the other candidate dopants, but is used here as a model system to demonstrate how substitutional doping can interact with intrinsic copper vacancies and modify the defect chemistry and electronic structure of Cu_2_S.

Although the S_Cu_ antisite defect exhibits the lowest defective formation energy among the configurations studied, it generates an excessively oxidized local chemical environment. Substituting S for Cu increases the local electronegativity, leading to significant charge depletion around neighboring atoms and overstabilizing surface sites, which reduces their reactivity. Such an overly oxidized environment is detrimental to the hydrogen evolution reaction (HER), as it limits the surface’s ability to efficiently adsorb and activate hydrogen intermediates. Furthermore, the defect-induced electronic states in this configuration are either too deep or too localized to participate effectively in catalytic charge-transfer processes.

In contrast, the V_Cu_ defect promotes a more balanced electronic redistribution. Removing a Cu atom creates undercoordinated sites and increases the local charge density around adjacent sulfur atoms, thereby facilitating more favorable hydrogen adsorption energetics. This moderate electronic activation improves interactions with reaction intermediates without overoxidizing the surface. Consequently, despite its slightly superior defective formation energy to that of S_Cu_ structure, the V_Cu_ structure is better suited for HER applications due to its more optimal electronic and chemical environment.

Under realistic growth conditions, a metallic dopant rarely integrates into a perfect lattice and instead interacts with pre-existing defects. In this study, we specifically focused on the V_Cu_+W defect complex to overcome the limited carrier activation caused by deep defect states in pristine Cu_2_S, which can be effectively tuned using an extrinsic defect model. A similar strategy was previously demonstrated in ref 49 for achieving self-doping in hybrid perovskites.

The rationale for this choice is that copper vacancies in Cu_2_S naturally act as acceptor-type defects, while W substitution provides an externally controllable means to modulate their thermodynamic stability and charge transition levels. The synergistic interaction between intrinsic V_Cu_ states and extrinsic W dopants stabilizes shallow acceptor levels near the valence band edge, effectively promoting **p**-type behavior without introducing deep trap states.^48^ Moreover, the partially filled 5*d* orbitals and high electronic polarizability of W contribute additional electronic states near the Fermi level, enhancing charge redistribution within the host lattice and further optimizing the material’s electronic properties.

Figure 7 presents the formation energies and thermodynamic charge transition levels of copper vacancies (V_Cu_) and tungsten-substituted copper vacancies (V_Cu_+W) in the Cu_2_S monolayer. As shown in Figure 7, both defects display negative slopes of Δ*H*_*f*_(*q*,*E*_*F*_) versus the Fermi level, indicative of shallow acceptor-like behavior. A marked difference is observed upon W incorporation: while the transition levels of isolated V_Cu_ defects lie deep in the band gap or outside the band edges, the V_Cu_+W complex introduces a prominent *ε*(−1/0) transition precisely at the valence band maximum (VBM). This alignment signals the formation of an efficient acceptor, enabling hole generation without an energetic penalty.

**Figure 7:**
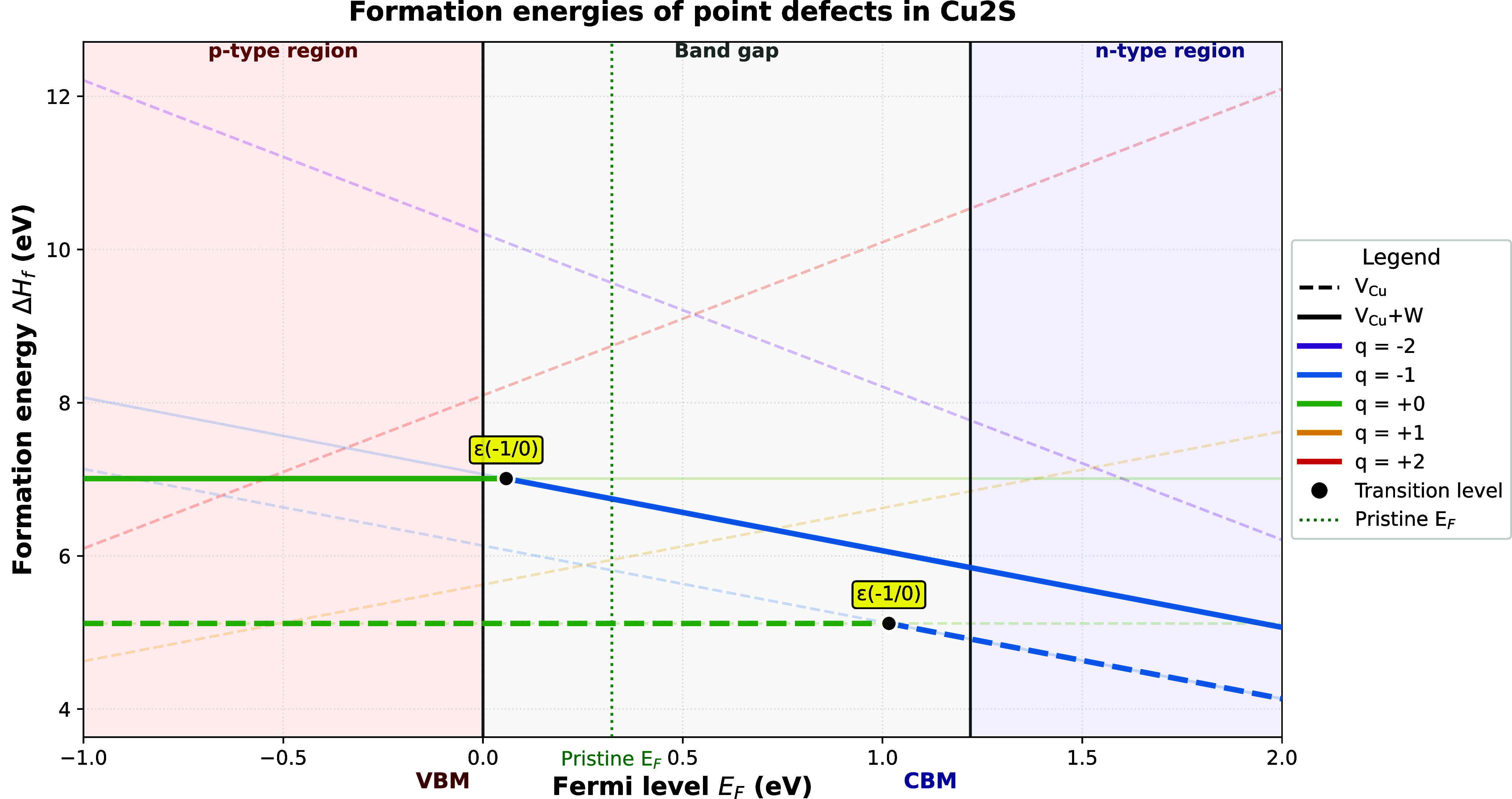
Formation energies Δ*H*_*f*_(*q*, *E*_*F*_) as a function of Fermi level *E*_*F*_ for copper vacancies (V_Cu_, dashed lines) and tungsten-substituted copper vacancies (V_Cu_+W, solid lines). All possible charge-state lines (*q* = −2, −1, 0, + 2) are shown in light colors (low opacity), while the stable segments—where a given charge state has the lowest formation energy—are highlighted with vivid, thick lines. Transition levels *ε*(*q*/*q*′) are marked by black circles; the *ε*(−1/0) transition of V_Cu_+W, located exactly at the valence-band maximum (VBM), is emphasized with a yellow box.

The overall distribution of transition levels, summarized in Figure 7, further demonstrates that W substitution strengthens the **p**-type character of Cu vacancies by stabilizing shallow acceptor states near the VBM. These acceptor levels effectively pin the Fermi level close to the valence band, increasing hole density at and near the surface. From a catalytic perspective, the **p**-type nature induced by the V_Cu_+W defect complex is highly advantageous for HER. The enhanced hole concentration facilitates charge transfer and promotes favorable hydrogen adsorption by optimizing interactions between surface active sites and H* intermediates. This approach has been successfully applied to improve HER performance in transition metal dichalcogenides.^50^

To evaluate the impact of W doping on the HER activity of the defective Cu_2_S monolayer, we investigated hydrogen adsorption on the surface. Several potential adsorption sites were tested (see Table S4 in the Supporting Information). Upon structural optimization, the preferred adsorption site shifts from the S–W bridge to the top of a sulfur atom, with the hydrogen atom tilting toward the neighboring tungsten. This configuration reflects a stabilized adsorption state influenced by both S and W coordination (see Figure 8).

**Figure 8:**
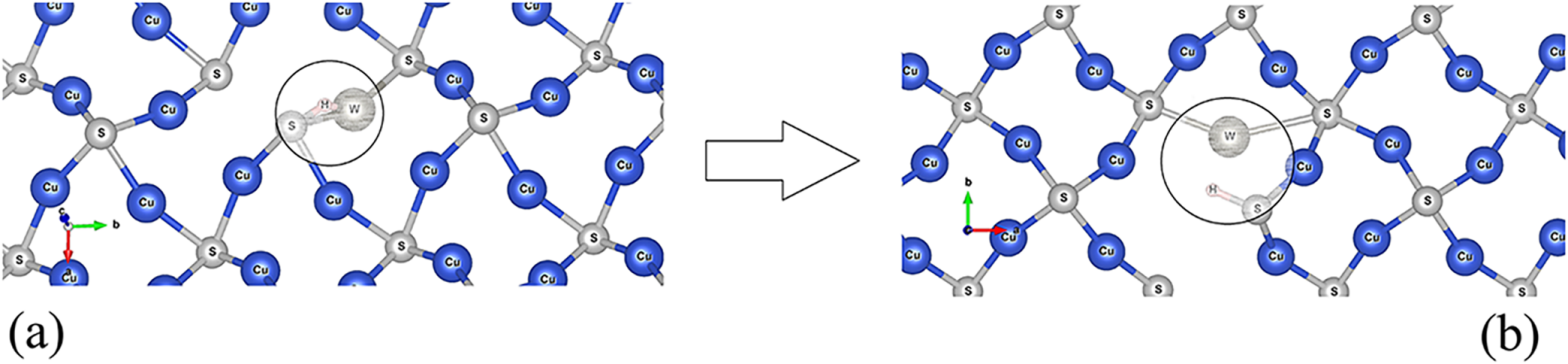
Optimized adsorption configurations of hydrogen on the S–W–Cu surface. (a) Initial adsorption at the S–W bridge site. (b) Stable adsorption at the top-S site, with H tilted downward toward the neighboring W atom. The structural transition from bridge to top-S is accompanied by a reorientation of the H–S bond, indicating residual interaction with the underlying tungsten. Cu, S, and W atoms are shown in blue, light gray, and dark gray, respectively; the adsorbed H atom is highlighted in light pink.

The hydrogen adsorption energy at this site was calculated to be −0.30 eV, indicating a moderately strong interaction between the hydrogen atom and the catalyst surface. After including zero-point energy and entropy corrections within the computational hydrogen electrode framework, the corresponding Gibbs free energy of hydrogen adsorption was estimated as Δ*G*_H*_ = −0.08 eV, close to thermoneutral conditions. This value represents a balanced hydrogen binding strength, favoring both proton–electron adsorption and subsequent hydrogen desorption, and is comparable to the optimal binding observed on Pt(111). The Δ*G*_H_ value for the pristine structure is calculated to be −0.66 eV, indicating a relatively strong binding between hydrogen and the surface. Such a negative value suggests that hydrogen adsorption is thermodynamically favorable; however, the binding is too strong, which may hinder the desorption step and thus limit the overall HER kinetics. In contrast, the introduction of copper-related defects significantly alters the adsorption behavior. The Δ*G*_H_ values for the S_Cu_ and V_Cu_ configurations are found to be 0.81 and 1.78 eV, respectively (see Table S5 in the Supporting Information), indicating highly unfavorable hydrogen adsorption.

The exchange current density (*j*_0_) is a key kinetic descriptor of electrocatalytic activity, corresponding to the reaction rate at zero overpotential. For the hydrogen evolution reaction, *j*_0_ can be estimated from the hydrogen adsorption free energy (Δ*G*_H_^*^) using a volcano-type relationship proposed by Nørskov et al.^42^

18 $$j_{0}\propto \exp\left(-\frac{\left|\Delta G_{H}^{*}\right|}{k_{B}T}\right)$$

This relation reflects the Sabatier principle, where optimal HER activity is achieved when Δ*G*_H_^*^ ≈ 0. However, it should be emphasized that this approach provides only a qualitative or semiquantitative estimate, as it relies on simplified kinetic assumptions and does not capture the full complexity of the reaction environment. Based on this relationship, the calculated value (*j*_0_ = 0.046 mA/cm^2^) suggests a favorable trend in HER activity. The near-thermoneutral Δ*G*_H_^*^ value indicates that the W-doped Cu_2_S system approaches the optimal adsorption regime, which is generally associated with improved HER activity.

### Correlation between HER Activity and Bonding Properties

To investigate the effect of tungsten doping on hydrogen adsorption, we performed charge density difference (CDD) and noncovalent interaction (NCI) analyses,^51,53^ which provide a clear electronic-level understanding of the hydrogen adsorption free energy, Δ*G*_H*_.^54^ Although the hydrogen atom primarily binds to the sulfur top site, the optimized geometry shows a tilted orientation toward the neighboring W atom, indicating a synergistic electronic coupling between the chalcogen site and the dopant transition metal center. Figure 9 presents both the CDD isosurfaces and the NCI representations.

**Figure 9:**
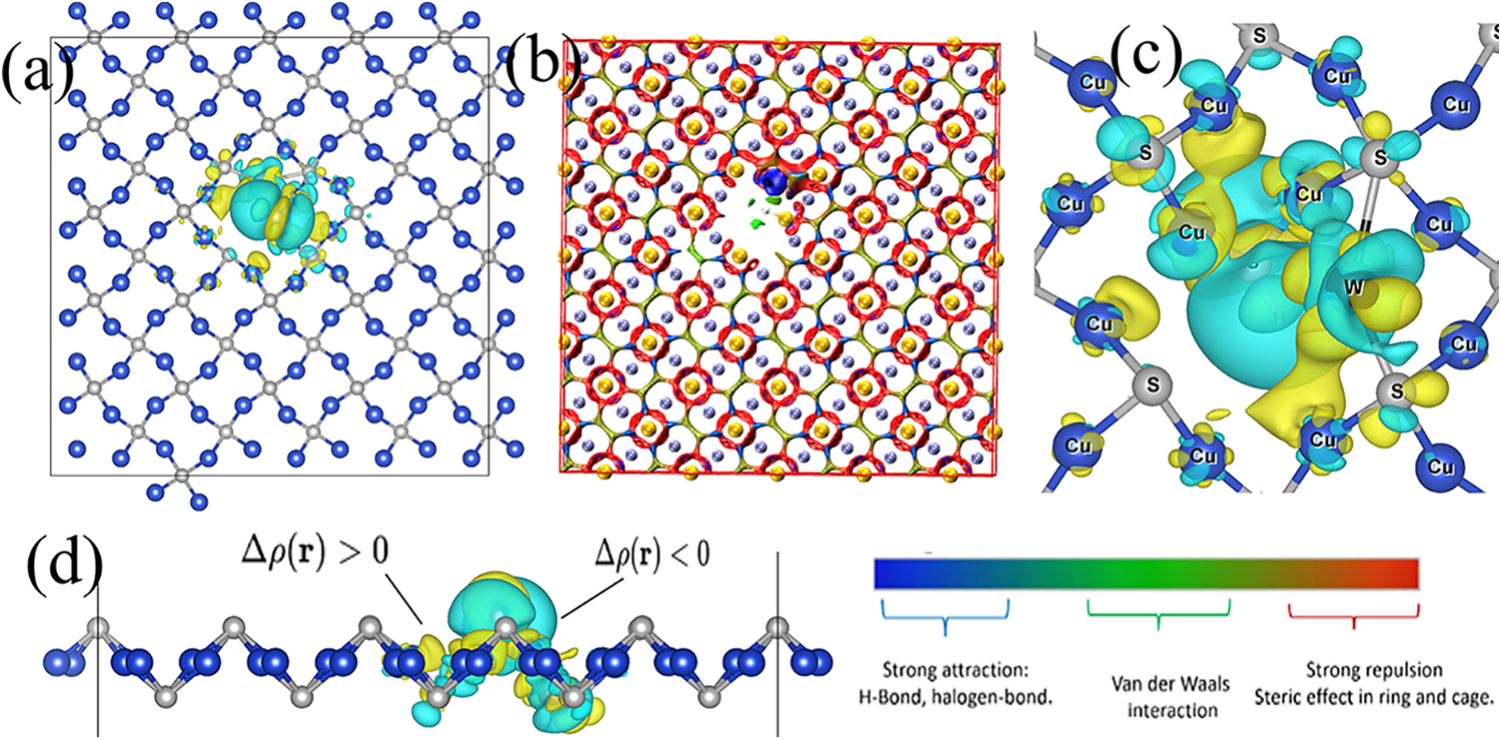
Charge density difference and noncovalent interaction (NCI) analyses for hydrogen adsorption on the Cu_2_S surface are shown in Figure 8. (a–c) Charge density difference (Δ*ρ*) plots, where yellow and cyan isosurfaces indicate regions of charge accumulation and depletion, respectively. Hydrogen adsorbs on the sulfur top site in a tilted orientation toward the neighboring tungsten atom, producing an asymmetric charge redistribution driven by the metal center. Charge accumulation at the H–S interface, coupled with depletion around the H atom, reflects partial charge transfer and polarization of the H*intermediate, which stabilizes hydrogen adsorption. (d) NCI isosurfaces, generated using the Critic2 code^51^ and colored according to the sign of λ_2_ρ, highlight attractive interactions (blue to green) between H* and S, along with van der Waals contributions (green). The absence of strong repulsive regions confirms the steric favorability of the tilted adsorption geometry. Together, the Δ*ρ* and NCI analyses indicate that hydrogen adsorption is governed by a synergistic effect: sulfur acts as the primary adsorption site, while tungsten serves as an electronic promoter. This combined interaction leads to an optimized hydrogen adsorption free energy (Δ*G*_H*_), favorable for the hydrogen evolution reaction.

The CDD analysis shows charge accumulation (Δ*ρ* > 0) at the H–S interface and charge depletion (Δ*ρ* < 0) near the hydrogen atom,^51^ revealing an asymmetric charge redistribution. Approximately 0.18 e is transferred from H* to the surface, polarizing the H–S bond. This polarization stabilizes the adsorbed hydrogen through orbital and electrostatic interactions, effectively lowering the adsorption energy. Notably, the charge redistribution also extends to the tungsten atom, demonstrating that W acts as an electronic facilitator, dispersing excess charge and preventing overbinding of hydrogen.^47^

The NCI analysis further complements these findings by identifying weak but stabilizing interactions not captured by the CDD. By plotting *sign*(λ_2_) × ρ­(**r**) versus *s*(ρ), we can differentiate interaction types relevant to the HER process. The NCI isosurfaces reveal a distinct region of attractive interactions (blue-to-green) between H*and S, corresponding to moderate directional interactions that stabilize the intermediate without forming strong covalent bonds.^52^ Together, these analyses highlight the cooperative role of sulfur and tungsten in optimizing hydrogen adsorption for efficient HER.

From Figure 9, it is evident that these interactions provide sufficient binding to anchor hydrogen on the surface while avoiding excessively negative Δ*G*_H*_ values that could impede H_2_ desorption. The additional green regions correspond to van der Waals interactions, which help smooth the potential energy surface along the HER pathway and further stabilize the adsorbed H. Hydrogen adsorption on the V_Cu_ + W structure is governed by a fine balance between charge transfer, bond polarization, and noncovalent stabilization, as revealed by the combined CDD and NCI analyses. Here, the sulfur atom serves as the primary adsorption site, while the adjacent tungsten atom modulates the electronic environment, ensuring that hydrogen binding is neither too strong nor too weak.

## Electronic Structure and Defect-Induced States

To elucidate the electronic origin of the defect-induced states and the nature of the shallow acceptor level, we analyzed the band structure and projected density of states (PDOS) of both the V_Cu_ and W-doped systems (V_Cu_ + W), as shown in Figure 10. For the pristine defect system (V_Cu_), the band structure exhibits a relatively clean band gap with no significant states near the Fermi level (E_F_), indicating the absence of electronically active shallow levels. However, upon introducing the W dopant, new electronic states emerge in the vicinity of E_F_. These states appear as nearly flat bands in the band structure, which is a characteristic signature of localized impurity levels arising from dopant atoms. The PDOS provides further insight into the origin of these states. A pronounced contribution from W-*d* orbitals is observed around the Fermi level, whereas the contributions from Cu and S atoms are significantly weaker in this region. This clearly demonstrates that the newly formed electronic states are predominantly derived from the W dopant. Importantly, these impurity states are located slightly above the valence band maximum (VBM) of the V_Cu_ case,^55^ indicating their shallow acceptor character. Their energetic proximity to the VBM suggests that they can be thermally activated, thereby promoting hole generation and enhancing **p**-type conductivity in the system. Furthermore, a partial hybridization between W-*d* and neighboring S-*p* states is observed, indicating a moderate interaction between the dopant and the host lattice. This hybridization contributes to the stabilization of the impurity states without pushing them deep into the band gap.^56^

**Figure 10:**
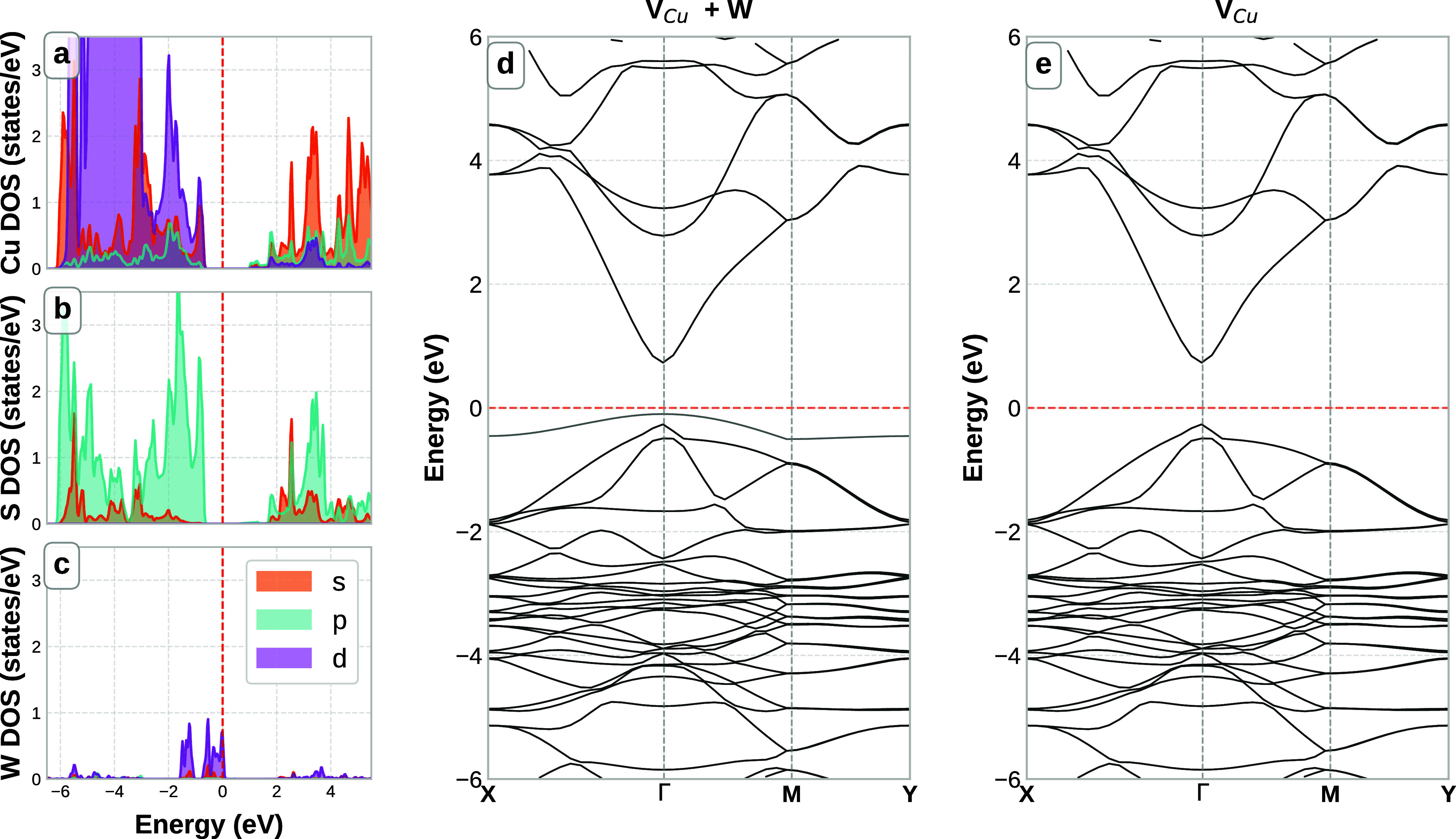
Electronic structure of the defective systems. left panel: projected density of states (PDOS) of (a) Cu, (b) S, and (c) W atoms of the V_Cu_ + W structure. Middle and right panels: band structures of (d) V_Cu_ and (e) V_Cu_ + W systems. The W-doped system exhibits nearly flat bands close to the Fermi level, indicating localized impurity states. The PDOS reveals that these states are mainly contributed by W-*d* orbitals.

## Conclusions

In this work, we systematically investigated the effects of intrinsic point defects and extrinsic W doping on the electronic structure and hydrogen evolution reaction activity of monolayer Cu_2_S using first-principles calculations. Several important conclusions emerge from our results. Four intrinsic defect configurations were considered: sulfur vacancies (V_S_), copper vacancies (V_Cu_), sulfur-on-copper antisites (S_Cu_), and copper-on-sulfur antisites (Cu_S_). Our thermodynamic analysis shows that the sulfur-on-copper antisite (S_Cu_) is the most energetically favorable intrinsic defect over a wide range of chemical potentials, indicating that it is likely to form preferentially during growth. In contrast, the copper vacancy (V_Cu_) becomes competitive under Cu-poor conditions and can also contribute significantly to the intrinsic defect landscape. Other defects, including V_S_ and Cu_S_, are energetically less favorable and are expected to occur in lower concentrations.

Importantly, we show that thermodynamic stability does not necessarily correlate with catalytic activity. Although S_Cu_ is the most stable defective structure, it generates an excessively oxidized local environment that suppresses hydrogen adsorption and limits catalytic activity. In contrast, V_Cu_ introduces under-coordinated sites and a more balanced charge redistribution, making it more relevant for hydrogen evolution despite its comparatively higher formation energy. From an electronic structure perspective, these intrinsic defects introduce defect states within the band gap with varying depth depending on the configuration. In particular, S_Cu_ induces states closer to the band edges, while other defects tend to introduce deeper localized states. Overall, these defect states are not optimal for efficient carrier generation and transport, which limits their direct contribution to HER performance.

Because of this limitation, we explored substitutional W doping at Cu sites as a strategy to further tailor the electronic properties. In this system, W is found to interact strongly with V_Cu_, forming a stable V_Cu_+W defect complex. This configuration modifies the electronic structure by stabilizing shallow acceptor-like states near the valence band edge while avoiding strongly localized trap states, thereby improving charge redistribution and carrier transport.

Catalytic calculations show that the V_Cu_+W defect complex yields a near-thermoneutral hydrogen adsorption free energy (Δ*G*_H*_ = −0.08 eV), which is generally considered favorable for HER activity. This behavior originates from the combined effect of vacancy-induced under-coordination and W-assisted electronic modulation around sulfur active sites, as supported by charge-density difference and noncovalent interaction analyses.

Overall, these results demonstrate that the catalytic performance of Cu_2_S is governed not only by the thermodynamic stability of intrinsic defects but also by their electronic activity and interaction with extrinsic dopants. The combined defect–dopant engineering strategy provides a viable route to tune carrier type, electronic conductivity, and surface reactivity in Cu-based chalcogenide monolayers, offering useful guidelines for the rational design of HER catalysts.

## Supplementary Material



## Data Availability

The data that support the findings of this study are available from the corresponding author upon reasonable request. Additionally, the data sets generated and/or analyzed during the current study are available in the Supporting Information.

## Notes

Further methodological details and complementary results that support the conclusions of the main manuscript; complete convergence tests and the thermodynamic framework for charged point-defect calculations in two-dimensional Cu_2_S; description of the two-dimensional Freysoldt–Neugebauer–Van de Walle correction scheme; definitions of chemical potentials; supercell convergence tests; defect formation-energy diagrams, charge transition levels, and hydrogen adsorption energies for the selected active sites on the V_Cu_+W structure (PDF)
