# Supplementary material for: Impact of Intrinsic Defects and Tungsten Doping on the Catalytic Properties of Two-Dimensional Cu2S
Source: ACS Omega. 2026 May 26;11(22):33202–15. doi: 10.1021/acsomega.6c03211 (PMC13261458; doi:10.1021/acsomega.6c03211)
Supplement: Supplementary file 1 [file ao6c03211_si_001.pdf]

Supporting Information
Impact of Intrinsic Defects and Tungsten Doping on the Catalytic Properties of Two-Dimensional Cu_2S

Tarik Ouahrani,[*,†,‡] David Dell'Angelo,[¶] Mohammed Benaissa,[§] Yasemin Oztekin Ciftci,[‖] Ángel Morales-García,[⊥] Michael Badawi,[#] and Daniel Errandonea[*,@]

†*École Supérieure en Sciences Appliquées, ESSA-Tlemcen, BB 165 RP Bel Horizon, Tlemcen 13000, Algeria.*

‡*Laboratoire de Physique Théorique, Université de Tlemcen, Tlemcen 13000, Algeria.*

¶*UCCS, CNRS, Université d'Artois, Faculté des Sciences Jean Perrin, Lens, 62307, Hauts-de-France, France*

§*Laboratory of Materials Discovery, Unit of Research Materials and Renewable Energies, LEPM-URMER. Université de Tlemcen 13000 Algeria.*

‖ *Department of Physics, Faculty of Science, Gazi University, 06500 Ankara, Turkey*

⊥*Departament de Ciéncia de Materials i Química Física & Institut de Química Teórica i Computacional (IQTCUB), Universitat de Barcelona, c/Martí i Franquès 1-11, 08028 Barcelona, Spain*

#*Université de Lorraine, CNRS, L2CM, F-54000 Nancy, France*

@*Departamento de Física Aplicada - Instituto de Ciencia de Materiales, Matter at High Pressure (MALTA) Consolider Team, Universidad de Valencia, Edificio de Investigación, C/Dr. Moliner 50, Burjassot, 46100, Valencia, Spain*

E-mail: tarik.ouahrani@univ-tlemcen.dz; daniel.errandonea@uv.es

Convergence tests

Figures S1, S2, and S3 show results from the tests carried out to determine the value of the Hubbard parameter U, the k-point grid, and the supercell used in calculations.

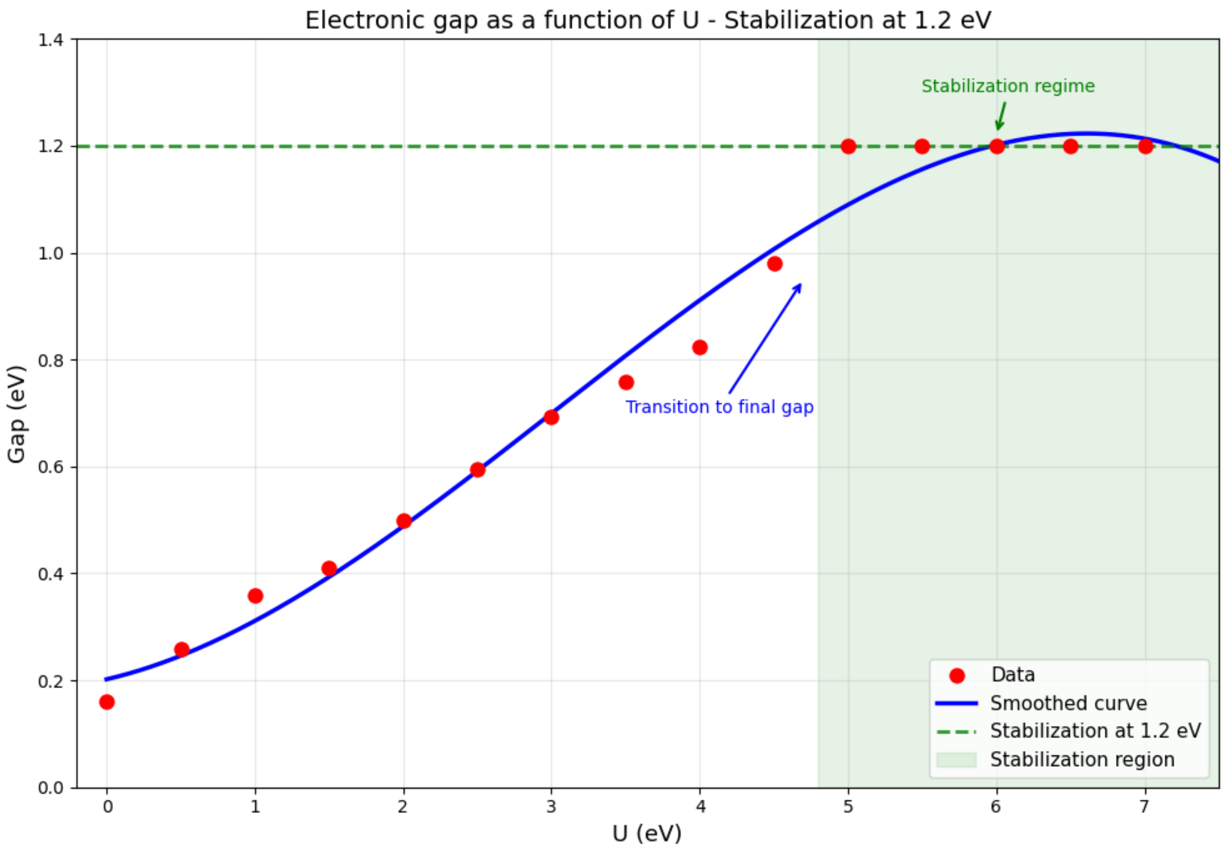

Figure S1 Hubbard parameter convergence tests for Cu atom within the DFT+U framework.

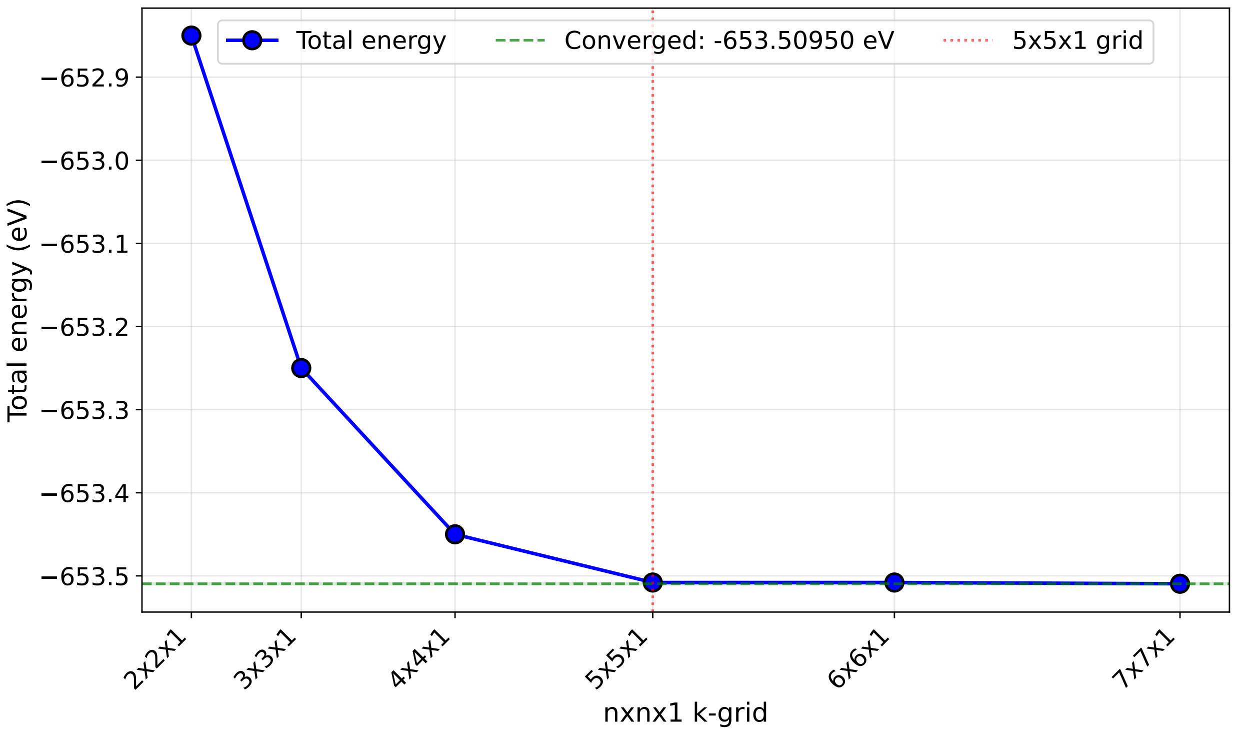

Figure S2 The convergence of the total energy of tetragonal Cu_2S with respect to supercell size is shown. Calculations were carried out using progressively larger in-plane supercells to assess finite-size effects. A $5 \times 5 \times 1$ supercell was ultimately chosen, as it provides satisfactory total-energy convergence while minimizing artificial interactions between periodic defects. The calculations were done at the GGA-PBE level and 1×1×1 grid.

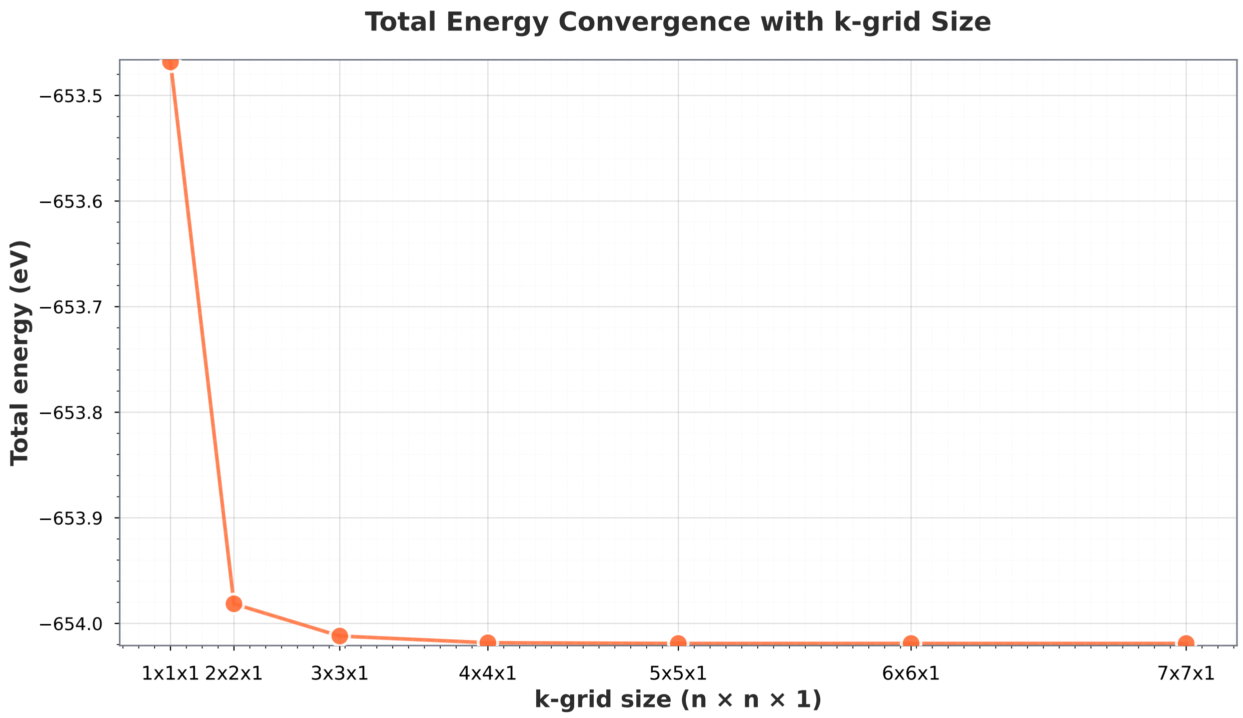

Figure S3 Total energy convergence as a function of k-point sampling for the defect $5 \times 5 \times 1$ supercell. A rapid decrease in energy variation is observed with increasing k-point density, reaching convergence within 1 meV at a $4{\times}4{\times}1$ grid. This confirms the adequacy of the selected k-point mesh for accurate defect energetics. The calculations were done at the GGA-PBE level.

Structural description and dynamical stability of the Cu_2S monolayer

Two crystallographic phases were identified for the Cu_2S monolayer. The first belongs to the orthorhombic space group *Pmma* (No. 51), while the second corresponds to the tetragonal $P42_12$ phase (No. 54), as illustrated in Figure S4.

In the *Pmma* structure, the lattice parameters satisfy $a \neq b \neq c$ with $\alpha = \beta = \gamma = 90°$, reflecting orthorhombic symmetry. This reduced symmetry results in an in-plane anisotropic atomic arrangement, which can give rise to direction-dependent electronic and mechanical properties.

By contrast, the $P42_12$ structure exhibits tetragonal symmetry, with $a = b \neq c$ and $\alpha = \beta = \gamma = 90°$. The presence of the 4_2 screw axis confers higher in-plane symmetry, rendering the x and y crystallographic directions equivalent. As a result, the in-plane electronic dispersion is expected to be more isotropic in this phase.

To assess the dynamical stability of the two Cu_2S configurations, phonon dispersion calculations were carried out, as shown in Figure S5. The phonon spectrum of the *Pmma* structure exhibits imaginary frequencies (negative values in the dispersion), indicating dynamical instability. This suggests that the orthorhombic phase does not correspond to a stable equilibrium structure for the Cu_2S monolayer.

Some of the observed structural differences between the two phases may stem from variations in crystallographic conventions (primitive versus conventional cells), axis reorientation, or minor symmetry reductions following structural relaxation. In contrast, the tetragonal $P42_12$ phase shows no imaginary phonon modes, confirming that it is the dynamically stable configuration of the Cu_2S monolayer. This phase was therefore used for all subsequent calculations.

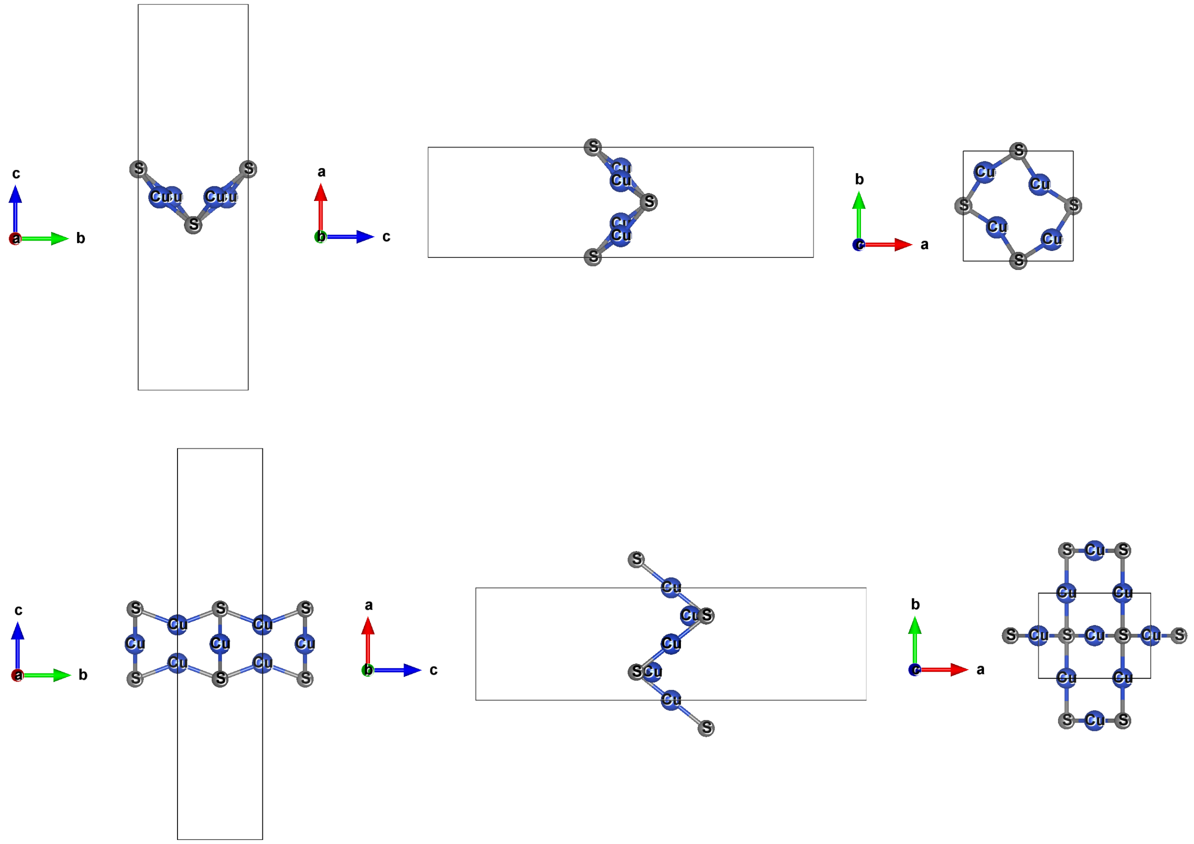

Figure S4 Illustration of the Cu_2S unit cells in two distinct crystallographic phases from selected viewing directions. The upper panels depict the tetragonal $P4 2_1 2$ phase, while the lower panels show the orthorhombic *Pmma* structure. Multiple crystallographic perspectives are provided for each phase to emphasize the atomic arrangement, symmetry elements, and local coordination environment. The comparison highlights the key structural differences between the two phases, particularly in lattice symmetry and the stacking of Cu–S atoms.

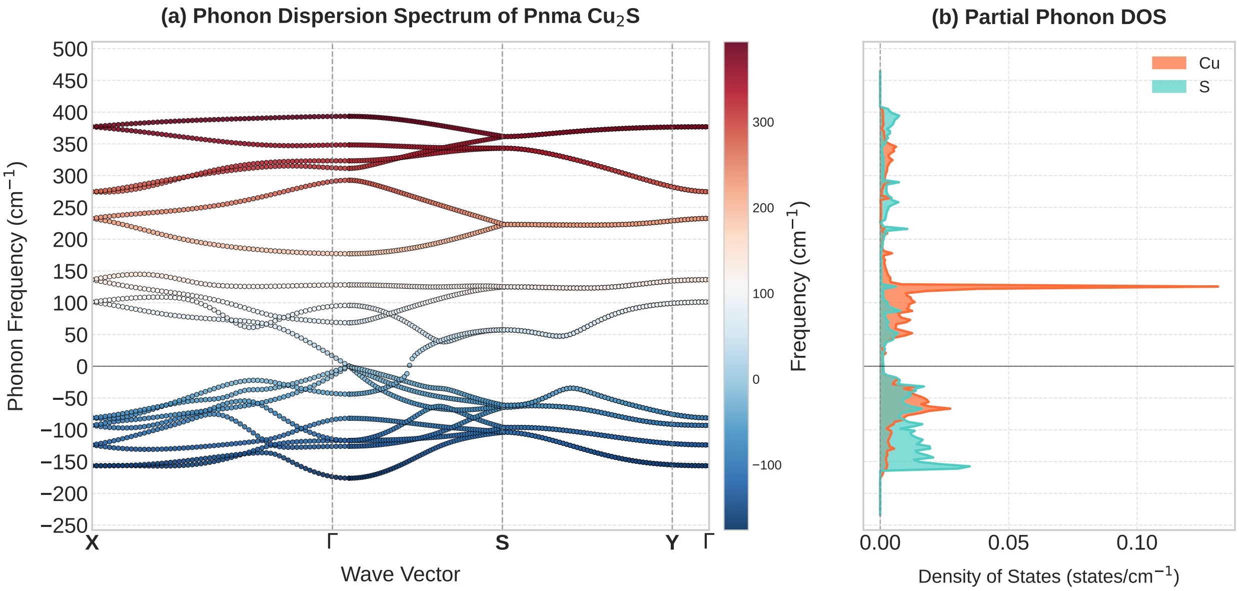

Figure S5 (a) Phonon dispersion and (b) phonon density of states of orthorhombic Cu_2S single layer

Electrostatic correction for charged defects

When modeling charged defects with periodic supercells, artificial electrostatic interactions can arise between repeated defect images, accompanied by an unphysical shift of the reference electrostatic potential. These finite-size effects are intrinsic to supercell calculations and are especially significant in two-dimensional systems due to reduced dielectric screening and the presence of vacuum regions.

In this study, the electronic structure was treated using the GGA+U method, which provides a more accurate description of the localized Cu 3d states and corrects the band-gap underestimation inherent to standard GGA. This improved band-gap representation is crucial for reliable defect-level alignment and for the precise calculation of charged-defect formation energies.

To remove the remaining spurious electrostatic contributions associated with charged defects, we employed a two-dimensional adaptation of the Freysoldt–Neugebauer–Van de Walle (FNV) correction scheme,[1] which is specifically designed for slab and low-dimensional

geometries. Within this approach, the total correction energy is written as

$$E_{\mathrm{corr}} = E_{\mathrm{PC}}^{\mathrm{2D}} + q\Delta V, \tag{S1}$$

where $E_{\mathrm{PC}}^{\mathrm{2D}}$ accounts for the long-range Coulomb interaction between a point charge and its periodic images in a two-dimensional system, and ΔV represents the potential alignment correction.

The potential alignment term ΔV was obtained by comparing the planar-averaged electrostatic potentials of the defective and pristine supercells,

$$\Delta V = \langle V_{\mathrm{defect}}(z) - V_{\mathrm{bulk}}(z) \rangle_{\mathrm{far}}\,, \tag{S2}$$

where the average is taken in a region sufficiently far from the defect (typically 5–7 Å away from the defect plane), where the electrostatic potential reaches a well-defined plateau.

For the $5 \times 5 \times 1$ supercells used in this study, the long-range electrostatic correction is given by

$$E_{\mathrm{PC}}^{\mathrm{2D}} = \frac{q^2}{2} \left(\frac{2\pi}{A\varepsilon_{\mathrm{2D}}} \right) L_z, \tag{S3}$$

where $A = (5a)^2 = 370.56$ Å^2 is the in-plane area of the supercell, L_z is the out-of-plane lattice parameter including the vacuum region (approximately 20 Å), and $\varepsilon_{\mathrm{2D}} = 4.2$ is the effective two-dimensional dielectric constant obtained from DFPT calculations within the GGA+U framework.

Additional information

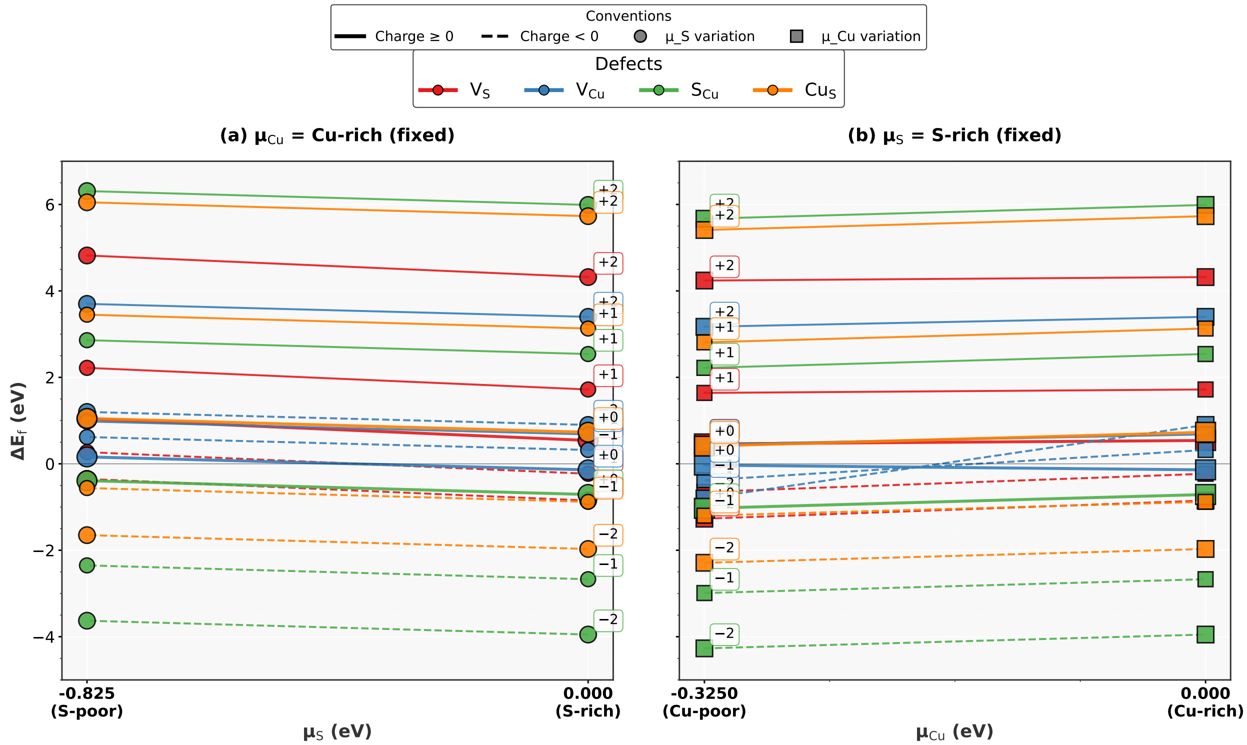

Figure S6 (a) Formation energies (ΔE_f) of intrinsic point defects in the Cu2S monolayer are plotted as a function of the Fermi level (E_F) referenced to the valence band maximum (VBM). Each straight line has a slope corresponding to the defect charge state q, covering values from $q = -2$ to $q = +2$ for sulfur vacancies (VS), copper vacancies (V_{Cu}), sulfur-on-copper antisites (S_{Cu}), and copper-on-sulfur antisites (Cu_{S}). Thicker solid lines indicate neutral defects ($q = 0$), while dashed lines represent negatively charged states. Thermodynamic charge transition levels $\varepsilon(q/q')$ are determined by the intersections of formation-energy lines for adjacent charge states. The shaded gray area marks the fundamental band gap of the Cu2S monolayer, with the VBM set to 0 eV. Calculations are performed under stoichiometric conditions ($\Delta\mu\mathrm{Cu} = \Delta\mu_{\mathrm{S}} = 0$). (b) Influence of chemical potential limits on defect formation energies. Panel (a) corresponds to Cu-rich conditions ($\Delta\mu_{\mathrm{Cu}} = 0$), while panel (b) shows S-rich conditions ($\Delta\mu_{\mathrm{S}} = 0$). Changes in chemical potentials shift the absolute formation energies but do not affect the slopes of the lines, highlighting that intrinsic defect chemistry predominantly governs Fermi-level pinning in the Cu_2S monolayer.

Table S1 Charge-state–dependent formation energies ΔE_f (eV) of intrinsic point defects in the Cu_2S monolayer under different chemical growth conditions. The table includes data for pristine, V_S, V_{Cu}, S_{Cu}, and Cu_S systems. For each system, the defect type, charge state (q), total number of electrons (NELECT), total DFT energy (TOTEN), valence-band maximum (VBM), conduction-band minimum (CBM), band gap ($E_g = E_{CBM} - E_{VBM}$), Fermi level (E_{Fermi}), and electrostatic finite-size correction (E_{corr}) applied to charged defects are reported.

Defect	q	NELECT	TOTEN (eV)	VBM (eV)	CBM (eV)	E_g (eV)	E_{Fermi} (eV)	E_{corr} (eV)
Pristine	-2	2002	-658.907	-2.4878	-0.3868	2.101	-2.1268	–
Pristine	-1	2001	-656.336	-2.7104	-0.5974	2.113	-2.7104	–
Pristine	0	2000	-653.509	-3.0985	-1.8795	1.219	-3.0132	–
Pristine	$+1$	1999	-650.340	-3.3189	-2.0949	1.224	-3.2951	–
Pristine	$+2$	1998	-646.926	-3.5396	-2.3076	1.232	-3.5345	–
V_S	-2	1996	-651.939	-2.4684	-1.2794	1.189	-2.1034	3.462
V_S	-1	1995	-649.371	-2.6505	-1.4705	1.180	-2.6488	0.865
V_S	0	1994	-646.580	-3.1200	-2.4060	0.714	-2.9928	0.000
V_S	$+1$	1993	-643.398	-3.3425	-2.4045	0.938	-3.3132	0.865
V_S	$+2$	1992	-639.968	-3.5636	-2.4236	1.140	-3.5528	3.462
V_{Cu}	-2	1985	-653.868	-3.0949	-2.4229	0.672	-2.1034	3.452
V_{Cu}	-1	1984	-651.303	-3.0949	-2.4229	0.672	-2.6488	0.870
V_{Cu}	0	1983	-648.391	-3.0949	-2.4229	0.672	-2.9928	0.000
V_{Cu}	$+1$	1982	-645.191	-3.0949	-2.4229	0.672	-3.3132	0.865
V_{Cu}	$+2$	1981	-641.749	-3.0949	-2.4229	0.672	-3.5528	3.452
S_{Cu}	-2	1991	-657.934	-2.1308	-1.2228	0.908	-2.1186	3.455
S_{Cu}	-1	1990	-655.657	-2.9057	-1.5847	1.321	-2.5981	0.862
S_{Cu}	0	1989	-652.684	-3.1251	-1.7731	1.352	-3.0993	0.000
S_{Cu}	$+1$	1988	-649.467	-3.3449	-1.9019	1.443	-3.3384	0.865
S_{Cu}	$+2$	1987	-646.014	-3.5653	-1.9733	1.812	-3.5727	3.455
Cu_S	-2	2013	-655.054	-1.6763	-1.3163	0.360	-1.6594	3.460
Cu_S	-1	2012	-653.163	-2.6445	-1.7805	0.864	-2.3371	0.865
Cu_S	0	2011	-650.436	-2.8655	-1.5495	1.316	-2.8657	0.000
Cu_S	$+1$	2010	-647.433	-3.0867	-1.6987	1.388	-3.1595	0.865
Cu_S	$+2$	2009	-644.126	-3.4873	-3.0673	0.420	-3.4652	3.460

Table S2 Variation in the number of atoms (Δn_{Cu}, Δn_S) and corresponding chemical potentials (μ_{Cu}, μ_S) used to compute the formation energies of point defects in the Cu_2S monolayer under Cu-rich and S-rich growth conditions. The chemical potentials are referenced to their elemental phases.

Defect	Δn_{Cu}	Δn_S	Growth condition	μ_{Cu} (eV)	μ_S (eV)
V_S	0	-1	Cu-rich	0.00	-0.825
V_S	0	-1	S-rich	-0.325	-0.165
V_{Cu}	-1	0	Cu-rich	0.00	-0.825
V_{Cu}	-1	0	S-rich	-0.325	-0.165
S_{Cu}	-1	$+1$	Cu-rich	0.00	-0.825
S_{Cu}	-1	$+1$	S-rich	-0.325	-0.165
Cu_S	$+1$	-1	Cu-rich	0.00	-0.825
Cu_S	$+1$	-1	S-rich	-0.325	-0.165

Table S3 Formation energies ΔE_f (eV) of point defects in the Cu_2S monolayer for all charge states q, calculated using charge-dependent VBM and Fermi-level energies, including electrostatic finite-size corrections. Cu-rich (S-rich) conditions correspond to the maximum (minimum) allowed chemical potential of Cu (S), while Cu-poor and S-poor denote the opposite limits. The chemical potentials have been updated according to experimental formation enthalpy.

Defect	q	Cu-rich	S-rich	Cu-poor	S-poor
V_S	-2	-1.41	-2.08	-0.99	-1.66
	-1	-0.15	-0.82	0.27	-0.40
	0	0.54	-0.13	0.96	0.29
	$+1$	1.66	0.99	2.08	1.41
	$+2$	4.17	3.50	4.59	3.92
V_{Cu}	-2	0.34	-1.86	0.70	-1.22
	-1	0.40	-0.80	0.76	-0.16
	0	-0.14	0.25	0.22	0.72
	$+1$	0.62	0.64	1.00	1.02
	$+2$	3.25	3.27	3.63	3.65
S_{Cu}	-2	-4.51	-4.89	-4.19	-4.57
	-1	-2.59	-2.97	-2.27	-2.65
	0	-0.71	-1.09	-0.39	-0.77
	$+1$	2.48	2.10	2.80	2.42
	$+2$	5.84	5.46	6.16	5.78
Cu_S	-2	-2.53	-2.91	-2.21	-2.59
	-1	-0.80	-1.18	-0.48	-0.86
	0	0.73	0.35	1.05	0.67
	$+1$	3.07	2.69	3.39	3.01
	$+2$	5.58	5.20	5.90	5.52

Table S4 Energy adsorption of the selected tested active sites on the V_{Cu}+W structure.

Case	E_{ads} (eV)
V_{Cu}	+1.22
W-top	+1.55
S-top	-0.12
Cu-top	+1.14
W–S-bridge to inclined S site	-0.30

Table S5 Hydrogen adsorption energy (E_{ads}) and Gibbs free energy of hydrogen adsorption (ΔG_{H}) for pristine and defective configurations.

System	E_{ads} (eV)	ΔG_{H} (eV)
Pristine	−0.88	−0.66
S_{Cu}	0.566	0.81
V_{Cu}	1.536	1.78

References

(1) C. Freysoldt, J. Neugebauer, C. G. Van de Walle, Fully Ab Initio Finite-Size Corrections for Charged-Defect Supercell Calculations, Phys. Rev. Lett. **102**, 016402 (2009).
